# Supplementary material for: Harnessing consumer wearable digital biomarkers for individualized recognition of postpartum depression using the All of Us Research Program dataset
Source: medRxiv. 2023 Oct 14:2023.10.13.23296965. Preprint. [Version 1] doi: 10.1101/2023.10.13.23296965 (PMC10593061; doi:10.1101/2023.10.13.23296965)
Supplement: Supplement 2 [file media-2.pdf]

Supplementary Table 7: OMOP concept IDs used in computational phenotyping of PPD and non-PPD cohorts.

Name	Concept Id	Variable	Variable class	Data source	Notes
Delivery by cesarean section for footling breech presentation	46273629	Cesarean section	Delivery	Conditions	
Delivery by cesarean section for flexed breech presentation	46273305	Cesarean section	Delivery	Conditions	
Delivery by cesarean section for breech presentation	46273304	Cesarean section	Delivery	Conditions	
Emergency lower segment cesarean section with inverted T incision	46270991	Cesarean section	Delivery	Conditions	
Other specified other caesarean delivery	44513733	Cesarean section	Delivery	Conditions	
Other specified elective caesarean delivery	44513729	Cesarean section	Delivery	Conditions	
Cesarean section through J shaped incision of uterus	42872493	Cesarean section	Delivery	Conditions	
Cesarean section through inverted T shaped incision of uterus	42872492	Cesarean section	Delivery	Conditions	
Triplet liveborn in hospital by cesarean section	42539210	Cesarean section	Delivery	Conditions	
Emergency lower segment cesarean section with bilateral tubal ligation	42537021	Cesarean section	Delivery	Conditions	
Elective lower segment cesarean section with bilateral tubal ligation	42536960	Cesarean section	Delivery	Conditions	
Emergency upper segment cesarean section with bilateral tubal ligation	42536954	Cesarean section	Delivery	Conditions	
Elective upper segment cesarean section with bilateral tubal ligation	42536952	Cesarean section	Delivery	Conditions	
Single liveborn born in hospital by cesarean section	40483521	Cesarean section	Delivery	Conditions	
Liveborn born in hospital by cesarean section	40483126	Cesarean section	Delivery	Conditions	
Twin liveborn born in hospital by cesarean section	40483101	Cesarean section	Delivery	Conditions	
Cesarean section w/o CC/MCC	38001486	Cesarean section	Delivery	Conditions	
Cesarean section w CC/MCC	38001485	Cesarean section	Delivery	Conditions	
Lower uterine segment cesarean section	37312440	Cesarean section	Delivery	Conditions	
Preterm delivery following Cesarean section	37110284	Cesarean section	Delivery	Conditions	
Born by emergency cesarean section	4250010	Cesarean section	Delivery	Conditions	
Vaginal cesarean section	4228344	Cesarean section	Delivery	Conditions	
Classical cesarean section	4223536	Cesarean section	Delivery	Conditions	
Born by elective cesarean section	4212794	Cesarean section	Delivery	Conditions	
Extraperitoneal cesarean section	4211824	Cesarean section	Delivery	Conditions	
Born by cesarean section	4192676	Cesarean section	Delivery	Conditions	
Placenta previa found before labor AND delivery by cesarean section without hemorrhage	4172142	Cesarean section	Delivery	Conditions	
Anesthesia for cesarean section	4171820	Cesarean section	Delivery	Conditions	
Emergency cesarean section	4167089	Cesarean section	Delivery	Conditions	
Abdominal delivery for shoulder dystocia	4130321	Cesarean section	Delivery	Conditions	
Emergency lower segment cesarean section	4127252	Cesarean section	Delivery	Conditions	
Delivered by cesarean delivery following previous cesarean delivery	4119336	Cesarean section	Delivery	Conditions	
Delivered by cesarean section - pregnancy at term	4118802	Cesarean section	Delivery	Conditions	
Elective cesarean section	4075182	Cesarean section	Delivery	Conditions	
Elective lower segment cesarean section	4075161	Cesarean section	Delivery	Conditions	
Elective upper segment cesarean section	4075160	Cesarean section	Delivery	Conditions	
Delivery by emergency cesarean section	4066112	Cesarean section	Delivery	Conditions	
Cesarean section - pregnancy at term	4066111	Cesarean section	Delivery	Conditions	
Cesarean section following previous cesarean section	4065739	Cesarean section	Delivery	Conditions	
Multiple delivery, all by cesarean section	4063163	Cesarean section	Delivery	Conditions	
Delivery by elective cesarean section	4061457	Cesarean section	Delivery	Conditions	
Emergency upper segment cesarean section	4032767	Cesarean section	Delivery	Conditions	
Cesarean section	4015701	Cesarean section	Delivery	Conditions	
Cesarean delivery only, following attempted vaginal delivery after previous cesarean delivery; including postpartum care	2110324	Cesarean section	Delivery	Conditions	
Cesarean delivery only, following attempted vaginal delivery after previous cesarean delivery	2110323	Cesarean section	Delivery	Conditions	
Cesarean delivery only; including postpartum care	2110317	Cesarean section	Delivery	Conditions	
Cesarean delivery only	2110316	Cesarean section	Delivery	Conditions	
Anesthesia for cesarean delivery following neuraxial labor analgesia/anesthesia (List separately in addition to code for primary procedure performed)	2101814	Cesarean section	Delivery	Conditions	
Anesthesia for cesarean delivery only	2101807	Cesarean section	Delivery	Conditions	
Neuraxial analgesia/anesthesia for labor ending in a cesarean delivery (includes any repeat subarachnoid needle placement and drug injection and/or any necessary replacement of an epidural catheter during labor)	2101016	Cesarean section	Delivery	Conditions	
Anesthesia for intraperitoneal procedures in lower abdomen including laparoscopy; cesarean section	2101013	Cesarean section	Delivery	Conditions	
Other cesarean section of unspecified type	2004802	Cesarean section	Delivery	Conditions	
Cesarean section of other specified type	2004789	Cesarean section	Delivery	Conditions	
Low cervical cesarean section	2004786	Cesarean section	Delivery	Conditions	
Cesarean delivery - delivered	437942	Cesarean section	Delivery	Conditions	
Deliveries by cesarean	193277	Cesarean section	Delivery	Conditions	
Delivery by cesarean section for footling breech presentation	46273629	Delivery record only (DELIV)	Delivery	Conditions	
Delivery by cesarean section for flexed breech presentation	46273305	Delivery record only (DELIV)	Delivery	Conditions	
Delivery by cesarean section for breech presentation	46273304	Delivery record only (DELIV)	Delivery	Conditions	
Vacuum assisted vaginal delivery	45757174	Delivery record only (DELIV)	Delivery	Conditions	
Other specified cephalic vaginal delivery with abnormal presentation of head at delivery without instrument	44513756	Delivery record only (DELIV)	Delivery	Conditions	
Other specified vacuum delivery	44513752	Delivery record only (DELIV)	Delivery	Conditions	
Other specified forceps cephalic delivery	44513747	Delivery record only (DELIV)	Delivery	Conditions	

Low forceps cephalic delivery	44513746	Delivery record only (DELIV)	Delivery	Conditions	
Mid forceps cephalic delivery NEC	44513745	Delivery record only (DELIV)	Delivery	Conditions	
High forceps cephalic delivery NEC	44513743	Delivery record only (DELIV)	Delivery	Conditions	
Other specified other breech delivery	44513740	Delivery record only (DELIV)	Delivery	Conditions	
Other specified breech extraction delivery	44513736	Delivery record only (DELIV)	Delivery	Conditions	
Multiple liveborn in hospital by vaginal delivery	42539267	Delivery record only (DELIV)	Delivery	Conditions	
Mid vacuum delivery	42535817	Delivery record only (DELIV)	Delivery	Conditions	
Outlet vacuum delivery	42535816	Delivery record only (DELIV)	Delivery	Conditions	
Born by mid-cavity forceps delivery	37310404	Delivery record only (DELIV)	Delivery	Conditions	
Born by low forceps delivery	37310393	Delivery record only (DELIV)	Delivery	Conditions	
Born by high forceps delivery	37310369	Delivery record only (DELIV)	Delivery	Conditions	
Single liveborn born in hospital by vaginal delivery	36713074	Delivery record only (DELIV)	Delivery	Conditions	
Vaginal delivery, medical personnel present	4331179	Delivery record only (DELIV)	Delivery	Conditions	
Forceps delivery with rotation of fetal head	4324549	Delivery record only (DELIV)	Delivery	Conditions	
Pinard maneuver	4294827	Delivery record only (DELIV)	Delivery	Conditions	
Trial forceps delivery	4290245	Delivery record only (DELIV)	Delivery	Conditions	
Mid forceps delivery	4266683	Delivery record only (DELIV)	Delivery	Conditions	
Kristeller maneuver	4250441	Delivery record only (DELIV)	Delivery	Conditions	
Born by breech delivery	4250009	Delivery record only (DELIV)	Delivery	Conditions	
Frank breech delivery	4244672	Delivery record only (DELIV)	Delivery	Conditions	
Bracht maneuver	4240325	Delivery record only (DELIV)	Delivery	Conditions	
Delivery by Malstrom's extraction	4234710	Delivery record only (DELIV)	Delivery	Conditions	
Partial breech delivery	4234421	Delivery record only (DELIV)	Delivery	Conditions	
Delivery by Kielland rotation	4232028	Delivery record only (DELIV)	Delivery	Conditions	
High forceps delivery	4231702	Delivery record only (DELIV)	Delivery	Conditions	
Partial breech extraction	4230533	Delivery record only (DELIV)	Delivery	Conditions	
Delivery by Malstrom's extraction with episiotomy	4223638	Delivery record only (DELIV)	Delivery	Conditions	
Footling breech delivery	4217642	Delivery record only (DELIV)	Delivery	Conditions	
Born by forceps delivery	4217586	Delivery record only (DELIV)	Delivery	Conditions	
Breech extraction	4213387	Delivery record only (DELIV)	Delivery	Conditions	
Spontaneous vertex delivery	4205240	Delivery record only (DELIV)	Delivery	Conditions	
Total breech delivery with forceps to aftercoming head	4204679	Delivery record only (DELIV)	Delivery	Conditions	
Delivery by vacuum extraction	4189205	Delivery record only (DELIV)	Delivery	Conditions	
Premature pregnancy delivered	4175637	Delivery record only (DELIV)	Delivery	Conditions	
Neville-Barnes forceps delivery	4173513	Delivery record only (DELIV)	Delivery	Conditions	
Abnormal delivery	4170626	Delivery record only (DELIV)	Delivery	Conditions	
Simpson's forceps delivery	4170152	Delivery record only (DELIV)	Delivery	Conditions	
Vaginal delivery with forceps including postpartum care	4167018	Delivery record only (DELIV)	Delivery	Conditions	
Brow delivery	4166775	Delivery record only (DELIV)	Delivery	Conditions	
Total breech extraction	4166247	Delivery record only (DELIV)	Delivery	Conditions	
Face delivery	4164221	Delivery record only (DELIV)	Delivery	Conditions	
Delivered by low forceps delivery	4156948	Delivery record only (DELIV)	Delivery	Conditions	
Deliveries by spontaneous breech delivery	4154615	Delivery record only (DELIV)	Delivery	Conditions	
Delivered by mid-cavity forceps delivery	4153284	Delivery record only (DELIV)	Delivery	Conditions	
Barton's forceps delivery	4147974	Delivery record only (DELIV)	Delivery	Conditions	
Midforceps delivery without rotation	4143647	Delivery record only (DELIV)	Delivery	Conditions	
Breech presentation, no version	4137400	Delivery record only (DELIV)	Delivery	Conditions	
Delivery of the after coming head	4130319	Delivery record only (DELIV)	Delivery	Conditions	
Instrumental delivery	4128031	Delivery record only (DELIV)	Delivery	Conditions	
Term pregnancy delivered	4127705	Delivery record only (DELIV)	Delivery	Conditions	
Mauriceau Smellie Veit maneuver	4127251	Delivery record only (DELIV)	Delivery	Conditions	
Lovset's maneuver	4127250	Delivery record only (DELIV)	Delivery	Conditions	
Outlet forceps delivery	4127249	Delivery record only (DELIV)	Delivery	Conditions	
Nonrotational forceps delivery	4127248	Delivery record only (DELIV)	Delivery	Conditions	
Born after precipitate delivery	4119050	Delivery record only (DELIV)	Delivery	Conditions	
Abnormal head presentation delivery	4118904	Delivery record only (DELIV)	Delivery	Conditions	
Normal delivery - occipitoanterior	4118903	Delivery record only (DELIV)	Delivery	Conditions	
Forceps delivery	4114637	Delivery record only (DELIV)	Delivery	Conditions	
Breech extraction with internal podalic version	4114636	Delivery record only (DELIV)	Delivery	Conditions	
Delivery by double application of forceps	4106406	Delivery record only (DELIV)	Delivery	Conditions	
Prague maneuver	4103850	Delivery record only (DELIV)	Delivery	Conditions	
Wigand-Martin maneuver	4102595	Delivery record only (DELIV)	Delivery	Conditions	
Delivery by Scanzoni maneuver	4100410	Delivery record only (DELIV)	Delivery	Conditions	
Delivery by vacuum extraction with episiotomy	4096145	Delivery record only (DELIV)	Delivery	Conditions	
Mid forceps delivery with episiotomy	4093774	Delivery record only (DELIV)	Delivery	Conditions	
Low forceps delivery	4088084	Delivery record only (DELIV)	Delivery	Conditions	
Face to pubes birth	4083524	Delivery record only (DELIV)	Delivery	Conditions	
Low forceps delivery with episiotomy	4075735	Delivery record only (DELIV)	Delivery	Conditions	
Low vacuum delivery	4075191	Delivery record only (DELIV)	Delivery	Conditions	
DeLee forceps cephalic delivery with rotation	4075190	Delivery record only (DELIV)	Delivery	Conditions	
Midforceps cephalic delivery with rotation	4075189	Delivery record only (DELIV)	Delivery	Conditions	
High forceps cephalic delivery with rotation	4075188	Delivery record only (DELIV)	Delivery	Conditions	

Forceps cephalic delivery	4075187	Delivery record only (DELIV)	Delivery	Conditions	
Vacuum delivery before full dilation of cervix	4075171	Delivery record only (DELIV)	Delivery	Conditions	
High vacuum delivery	4075170	Delivery record only (DELIV)	Delivery	Conditions	
Barton forceps cephalic delivery with rotation	4075169	Delivery record only (DELIV)	Delivery	Conditions	
Assisted breech delivery	4075168	Delivery record only (DELIV)	Delivery	Conditions	
Breech extraction delivery with version	4075165	Delivery record only (DELIV)	Delivery	Conditions	
Piper forceps delivery	4073438	Delivery record only (DELIV)	Delivery	Conditions	
Trial of vacuum delivery	4073424	Delivery record only (DELIV)	Delivery	Conditions	
Spontaneous breech delivery	4073422	Delivery record only (DELIV)	Delivery	Conditions	
Non-manipulative cephalic vaginal delivery with abnormal presentation of head at delivery without instrument	4071630	Delivery record only (DELIV)	Delivery	Conditions	
Deliveries by destructive operation	4066113	Delivery record only (DELIV)	Delivery	Conditions	
Delivery by combination of forceps and vacuum extractor	4065737	Delivery record only (DELIV)	Delivery	Conditions	
Piper forceps delivery by application to aftercoming head	4064824	Delivery record only (DELIV)	Delivery	Conditions	
Multiple delivery, all by forceps and vacuum extractor	4063162	Delivery record only (DELIV)	Delivery	Conditions	
Normal delivery but ante- or post- natal conditions present	4063160	Delivery record only (DELIV)	Delivery	Conditions	
Delivered by mid-cavity forceps with rotation	4062136	Delivery record only (DELIV)	Delivery	Conditions	
High forceps delivery with episiotomy	4035778	Delivery record only (DELIV)	Delivery	Conditions	
Complete breech delivery	4034145	Delivery record only (DELIV)	Delivery	Conditions	
Burns Marshall maneuver	4032766	Delivery record only (DELIV)	Delivery	Conditions	
Groin traction at breech delivery	4032764	Delivery record only (DELIV)	Delivery	Conditions	
Forceps application to aftercoming head	4032762	Delivery record only (DELIV)	Delivery	Conditions	
Forceps delivery, face to pubes	4032761	Delivery record only (DELIV)	Delivery	Conditions	
Partial breech delivery with forceps to aftercoming head	4023797	Delivery record only (DELIV)	Delivery	Conditions	
Normal birth	4014720	Delivery record only (DELIV)	Delivery	Conditions	
Postmature pregnancy delivered	4011047	Delivery record only (DELIV)	Delivery	Conditions	
Threatened premature labor - delivered	3186670	Delivery record only (DELIV)	Delivery	Conditions	
Vaginal delivery only, after previous cesarean delivery (with or without episiotomy and/or forceps); including postpartum care	2110321	Delivery record only (DELIV)	Delivery	Conditions	
Routine obstetric care including antepartum care, vaginal delivery (with or without episiotomy, and/or forceps) and postpartum care, after previous cesarean delivery	2110319	Delivery record only (DELIV)	Delivery	Conditions	
Vaginal delivery only (with or without episiotomy and/or forceps); including postpartum care	2110309	Delivery record only (DELIV)	Delivery	Conditions	
Vaginal delivery only (with or without episiotomy and/or forceps)	2110308	Delivery record only (DELIV)	Delivery	Conditions	
Routine obstetric care including antepartum care, vaginal delivery (with or without episiotomy, and/or forceps) and postpartum care	2110307	Delivery record only (DELIV)	Delivery	Conditions	
Other specified instrumental delivery	2004731	Delivery record only (DELIV)	Delivery	Conditions	
Other vacuum extraction	2004730	Delivery record only (DELIV)	Delivery	Conditions	
Other total breech extraction	2004726	Delivery record only (DELIV)	Delivery	Conditions	
Other partial breech extraction	2004724	Delivery record only (DELIV)	Delivery	Conditions	
Other high forceps operation	2004710	Delivery record only (DELIV)	Delivery	Conditions	
Other mid forceps operation	2004707	Delivery record only (DELIV)	Delivery	Conditions	
Forceps, vacuum, and breech delivery	2004702	Delivery record only (DELIV)	Delivery	Conditions	
Vacuum extractor delivery - delivered	442069	Delivery record only (DELIV)	Delivery	Conditions	
Delivery normal	441641	Delivery record only (DELIV)	Delivery	Conditions	
Elderly primigravida - delivered	440794	Delivery record only (DELIV)	Delivery	Conditions	
Deliveries by breech extraction	440793	Delivery record only (DELIV)	Delivery	Conditions	
Deliveries by vacuum extractor	440790	Delivery record only (DELIV)	Delivery	Conditions	
Triplet pregnancy - delivered	440462	Delivery record only (DELIV)	Delivery	Conditions	
Quadruplet pregnancy - delivered	438481	Delivery record only (DELIV)	Delivery	Conditions	
Breech extraction - delivered	436767	Delivery record only (DELIV)	Delivery	Conditions	
Forceps delivery - delivered	435022	Delivery record only (DELIV)	Delivery	Conditions	
Twin pregnancy - delivered	435018	Delivery record only (DELIV)	Delivery	Conditions	
Grand multiparity - delivered	434110	Delivery record only (DELIV)	Delivery	Conditions	
Mother delivered	433260	Delivery record only (DELIV)	Delivery	Conditions	
Breech presentation - delivered	73537	Delivery record only (DELIV)	Delivery	Conditions	
Triplet liveborn in hospital by cesarean section	42539210	Delivery record only (DELIV)	Delivery	Conditions	
Twin liveborn born in hospital by cesarean section	40483101	Delivery record only (DELIV)	Delivery	Conditions	
Multiple delivery, all by cesarean section	4063163	Delivery record only (DELIV)	Delivery	Conditions	
Delayed delivery second twin - delivered	4064559	Delivery record only (DELIV)	Delivery	Conditions	
Locked twins - delivered	442422	Delivery record only (DELIV)	Delivery	Conditions	
Multiple pregnancy with malpresentation - delivered	442082	Delivery record only (DELIV)	Delivery	Conditions	
Emergency lower segment cesarean section with inverted T incision	46270991	Delivery record only (DELIV)	Delivery	Conditions	
Other specified other caesarean delivery	44513733	Delivery record only (DELIV)	Delivery	Conditions	
Other specified elective caesarean delivery	44513729	Delivery record only (DELIV)	Delivery	Conditions	
Cesarean section through J shaped incision of uterus	42872493	Delivery record only (DELIV)	Delivery	Conditions	
Cesarean section through inverted T shaped incision of uterus	42872492	Delivery record only (DELIV)	Delivery	Conditions	
Emergency lower segment cesarean section with bilateral tubal ligation	42537021	Delivery record only (DELIV)	Delivery	Conditions	
Elective lower segment cesarean section with bilateral tubal ligation	42536960	Delivery record only (DELIV)	Delivery	Conditions	
Emergency upper segment cesarean section with bilateral tubal ligation	42536954	Delivery record only (DELIV)	Delivery	Conditions	
Elective upper segment cesarean section with bilateral tubal ligation	42536952	Delivery record only (DELIV)	Delivery	Conditions	
Single liveborn born in hospital by cesarean section	40483521	Delivery record only (DELIV)	Delivery	Conditions	
Liveborn born in hospital by cesarean section	40483126	Delivery record only (DELIV)	Delivery	Conditions	

Cesarean section w/o CC/MCC	38001486	Delivery record only (DELIV)	Delivery	Conditions	
Cesarean section w CC/MCC	38001485	Delivery record only (DELIV)	Delivery	Conditions	
Lower uterine segment cesarean section	37312440	Delivery record only (DELIV)	Delivery	Conditions	
Preterm delivery following Cesarean section	37110284	Delivery record only (DELIV)	Delivery	Conditions	
Born by emergency cesarean section	4250010	Delivery record only (DELIV)	Delivery	Conditions	
Vaginal cesarean section	4228344	Delivery record only (DELIV)	Delivery	Conditions	
Classical cesarean section	4223536	Delivery record only (DELIV)	Delivery	Conditions	
Born by elective cesarean section	4212794	Delivery record only (DELIV)	Delivery	Conditions	
Extraperitoneal cesarean section	4211824	Delivery record only (DELIV)	Delivery	Conditions	
Born by cesarean section	4192676	Delivery record only (DELIV)	Delivery	Conditions	
Placenta previa found before labor AND delivery by cesarean section without hemorrhage	4172142	Delivery record only (DELIV)	Delivery	Conditions	
Anesthesia for cesarean section	4171820	Delivery record only (DELIV)	Delivery	Conditions	
Emergency cesarean section	4167089	Delivery record only (DELIV)	Delivery	Conditions	
Abdominal delivery for shoulder dystocia	4130321	Delivery record only (DELIV)	Delivery	Conditions	
Emergency lower segment cesarean section	4127252	Delivery record only (DELIV)	Delivery	Conditions	
Delivered by cesarean delivery following previous cesarean delivery	4119336	Delivery record only (DELIV)	Delivery	Conditions	
Delivered by cesarean section - pregnancy at term	4118802	Delivery record only (DELIV)	Delivery	Conditions	
Elective cesarean section	4075182	Delivery record only (DELIV)	Delivery	Conditions	
Elective lower segment cesarean section	4075161	Delivery record only (DELIV)	Delivery	Conditions	
Elective upper segment cesarean section	4075160	Delivery record only (DELIV)	Delivery	Conditions	
Delivery by emergency cesarean section	4066112	Delivery record only (DELIV)	Delivery	Conditions	
Cesarean section - pregnancy at term	4066111	Delivery record only (DELIV)	Delivery	Conditions	
Cesarean section following previous cesarean section	4065739	Delivery record only (DELIV)	Delivery	Conditions	
Delivery by elective cesarean section	4061457	Delivery record only (DELIV)	Delivery	Conditions	
Emergency upper segment cesarean section	4032767	Delivery record only (DELIV)	Delivery	Conditions	
Cesarean section	4015701	Delivery record only (DELIV)	Delivery	Conditions	
Cesarean delivery only, following attempted vaginal delivery after previous cesarean delivery; including postpartum care	2110324	Delivery record only (DELIV)	Delivery	Conditions	
Cesarean delivery only, following attempted vaginal delivery after previous cesarean delivery	2110323	Delivery record only (DELIV)	Delivery	Conditions	
Cesarean delivery only; including postpartum care	2110317	Delivery record only (DELIV)	Delivery	Conditions	
Cesarean delivery only	2110316	Delivery record only (DELIV)	Delivery	Conditions	
Anesthesia for cesarean delivery following neuraxial labor analgesia/anesthesia (List separately in addition to code for primary procedure performed)	2101814	Delivery record only (DELIV)	Delivery	Conditions	
Anesthesia for cesarean delivery only	2101807	Delivery record only (DELIV)	Delivery	Conditions	
Neuraxial analgesia/anesthesia for labor ending in a cesarean delivery (includes any repeat subarachnoid needle placement and drug injection and/or any necessary replacement of an epidural catheter during labor)	2101016	Delivery record only (DELIV)	Delivery	Conditions	
Anesthesia for intraperitoneal procedures in lower abdomen including laparoscopy; cesarean section	2101013	Delivery record only (DELIV)	Delivery	Conditions	
Other cesarean section of unspecified type	2004802	Delivery record only (DELIV)	Delivery	Conditions	
Cesarean section of other specified type	2004789	Delivery record only (DELIV)	Delivery	Conditions	
Low cervical cesarean section	2004786	Delivery record only (DELIV)	Delivery	Conditions	
Cesarean delivery - delivered	437942	Delivery record only (DELIV)	Delivery	Conditions	
Deliveries by cesarean	193277	Delivery record only (DELIV)	Delivery	Conditions	
Cystocele - delivered with postpartum complication	4129700	Delivery record only (DELIV)	Delivery	Conditions	
Hemorrhoids in pregnancy and the puerperium - delivered with postnatal complication	4066133	Delivery record only (DELIV)	Delivery	Conditions	
Puerperal septicemia - delivered with postnatal complication	4066124	Delivery record only (DELIV)	Delivery	Conditions	
Puerperal peritonitis - delivered with postnatal complication	4066122	Delivery record only (DELIV)	Delivery	Conditions	
Puerperal endometritis - delivered with postnatal complication	4065749	Delivery record only (DELIV)	Delivery	Conditions	
Obstetric anesthesia with central nervous system complications - delivered	4065622	Delivery record only (DELIV)	Delivery	Conditions	
Obstetric anesthesia with cardiac complications - delivered	4065621	Delivery record only (DELIV)	Delivery	Conditions	
Secondary postpartum hemorrhage - delivered with postnatal problem	4065618	Delivery record only (DELIV)	Delivery	Conditions	
Rupture of uterus during and after labor - delivered with postnatal problem	4064960	Delivery record only (DELIV)	Delivery	Conditions	
Hypertonic uterine inertia - delivered	4064834	Delivery record only (DELIV)	Delivery	Conditions	
Persistent occipitoposterior or occipitoanterior position - delivered	4064426	Delivery record only (DELIV)	Delivery	Conditions	
Fetus with drug damage - delivered	4064177	Delivery record only (DELIV)	Delivery	Conditions	
Fetus with viral damage via mother - delivered	4064174	Delivery record only (DELIV)	Delivery	Conditions	
Fetus with hereditary disease - delivered	4064171	Delivery record only (DELIV)	Delivery	Conditions	
Fetal distress - delivered	4063305	Delivery record only (DELIV)	Delivery	Conditions	
Fetus with damage due to intrauterine contraceptive device - delivered	4063296	Delivery record only (DELIV)	Delivery	Conditions	
Transverse lie - delivered	4063171	Delivery record only (DELIV)	Delivery	Conditions	
Glycosuria during pregnancy - delivered	4063028	Delivery record only (DELIV)	Delivery	Conditions	
Fatigue during pregnancy - delivered with postnatal complication	4062572	Delivery record only (DELIV)	Delivery	Conditions	
Fatigue during pregnancy - delivered	4062571	Delivery record only (DELIV)	Delivery	Conditions	
Hemorrhoids in pregnancy and the puerperium - delivered	4062268	Delivery record only (DELIV)	Delivery	Conditions	
Obstetric anesthesia with pulmonary complications - delivered with postnatal problem	4062116	Delivery record only (DELIV)	Delivery	Conditions	
Obstetric anesthesia with pulmonary complications - delivered	4062115	Delivery record only (DELIV)	Delivery	Conditions	
Puerperal salpingitis - delivered with postnatal complication	4061465	Delivery record only (DELIV)	Delivery	Conditions	
Obstetric anesthesia with central nervous system complication - delivered with postnatal problem	4061349	Delivery record only (DELIV)	Delivery	Conditions	
Obstetric anesthesia with cardiac complications - delivered with postnatal problem	4061346	Delivery record only (DELIV)	Delivery	Conditions	
Prolonged artificial rupture of membranes - delivered	4060695	Delivery record only (DELIV)	Delivery	Conditions	

Fetus with radiation damage - delivered	4060675	Delivery record only (DELIV)	Delivery	Conditions	
Uterine scar from previous surgery in pregnancy, childbirth and the puerperium - delivered	4060558	Delivery record only (DELIV)	Delivery	Conditions	
Brow presentation - delivered	4060540	Delivery record only (DELIV)	Delivery	Conditions	
Face presentation - delivered	4060539	Delivery record only (DELIV)	Delivery	Conditions	
Herpes gestationis - delivered	4060301	Delivery record only (DELIV)	Delivery	Conditions	
Umbilical cord tight around neck - delivered	4060157	Delivery record only (DELIV)	Delivery	Conditions	
Oblique lie - delivered	4059757	Delivery record only (DELIV)	Delivery	Conditions	
Eclampsia with postnatal complication	4058536	Delivery record only (DELIV)	Delivery	Conditions	
Antepartum hemorrhage with uterine leiomyoma - delivered	4058526	Delivery record only (DELIV)	Delivery	Conditions	
Antepartum hemorrhage with trauma - delivered	4058524	Delivery record only (DELIV)	Delivery	Conditions	
Glycosuria during pregnancy - delivered with postnatal complication	4058111	Delivery record only (DELIV)	Delivery	Conditions	
Herpes gestationis - delivered with postnatal complication	4058110	Delivery record only (DELIV)	Delivery	Conditions	
Rectocele - delivered with postpartum complication	4028632	Delivery record only (DELIV)	Delivery	Conditions	
Failed trial of labor - delivered	443521	Delivery record only (DELIV)	Delivery	Conditions	
Papyraceous fetus - delivered	443324	Delivery record only (DELIV)	Delivery	Conditions	
Obstetric non-purulent mastitis - delivered	443294	Delivery record only (DELIV)	Delivery	Conditions	
Antepartum hemorrhage with coagulation defect - delivered	443133	Delivery record only (DELIV)	Delivery	Conditions	
Pelvic soft tissue abnormality in pregnancy, childbirth and the puerperium - delivered	442829	Delivery record only (DELIV)	Delivery	Conditions	
Fetus with central nervous system malformation - delivered	442828	Delivery record only (DELIV)	Delivery	Conditions	
Obstetric pyemic and septic pulmonary embolism - delivered	442420	Delivery record only (DELIV)	Delivery	Conditions	
Puerperal cerebrovascular disorder - delivered	442419	Delivery record only (DELIV)	Delivery	Conditions	
Puerperal cerebrovascular disorder - delivered with postnatal complication	442418	Delivery record only (DELIV)	Delivery	Conditions	
Placental polyp - delivered with postnatal complication	442090	Delivery record only (DELIV)	Delivery	Conditions	
Obstetric nipple infection - delivered	442089	Delivery record only (DELIV)	Delivery	Conditions	
Obstetric laceration of cervix - delivered	442080	Delivery record only (DELIV)	Delivery	Conditions	
Obstetric shock - delivered	442072	Delivery record only (DELIV)	Delivery	Conditions	
Maternal hypotension syndrome - delivered	442070	Delivery record only (DELIV)	Delivery	Conditions	
Varicose veins of legs in pregnancy and the puerperium - delivered	442059	Delivery record only (DELIV)	Delivery	Conditions	
Amniotic fluid pulmonary embolism - delivered	442054	Delivery record only (DELIV)	Delivery	Conditions	
Obstetric blood-clot pulmonary embolism - delivered	442053	Delivery record only (DELIV)	Delivery	Conditions	
Obstetric air pulmonary embolism - delivered with postnatal complication	442052	Delivery record only (DELIV)	Delivery	Conditions	
Obstetric air pulmonary embolism - delivered	442049	Delivery record only (DELIV)	Delivery	Conditions	
Obstetric pyemic and septic pulmonary embolism - delivered with postnatal complication	442048	Delivery record only (DELIV)	Delivery	Conditions	
Failed mechanical induction - delivered	441924	Delivery record only (DELIV)	Delivery	Conditions	
Antenatal deep vein thrombosis - delivered	441649	Delivery record only (DELIV)	Delivery	Conditions	
Prolapse of cord - delivered	441645	Delivery record only (DELIV)	Delivery	Conditions	
Maternal distress - delivered with postnatal problem	441643	Delivery record only (DELIV)	Delivery	Conditions	
History of recurrent miscarriage - delivered	441630	Delivery record only (DELIV)	Delivery	Conditions	
Cervical incompetence - delivered	441369	Delivery record only (DELIV)	Delivery	Conditions	
Postpartum coagulation defects - delivered with postnatal problem	441363	Delivery record only (DELIV)	Delivery	Conditions	
Cracked nipple in pregnancy, the puerperium or lactation - delivered with postnatal complication	440481	Delivery record only (DELIV)	Delivery	Conditions	
Prolonged first stage - delivered	440475	Delivery record only (DELIV)	Delivery	Conditions	
Post-term pregnancy - delivered	439894	Delivery record only (DELIV)	Delivery	Conditions	
Superficial thrombophlebitis in pregnancy and the puerperium - delivered with postnatal complication	439095	Delivery record only (DELIV)	Delivery	Conditions	
Superficial thrombophlebitis in pregnancy and the puerperium - delivered	439094	Delivery record only (DELIV)	Delivery	Conditions	
Cord tangled with compression - delivered	439092	Delivery record only (DELIV)	Delivery	Conditions	
Severe pre-eclampsia - delivered with postnatal complication	439077	Delivery record only (DELIV)	Delivery	Conditions	
Severe pre-eclampsia - delivered	438490	Delivery record only (DELIV)	Delivery	Conditions	
Precipitate labor - delivered	438220	Delivery record only (DELIV)	Delivery	Conditions	
Amniotic cavity infection - delivered	437936	Delivery record only (DELIV)	Delivery	Conditions	
Fetal intrauterine distress first noted during labor AND/OR delivery in liveborn infant	437098	Delivery record only (DELIV)	Delivery	Conditions	
Obstetric nipple infection - delivered with postnatal complication	437055	Delivery record only (DELIV)	Delivery	Conditions	
Varicose veins of perineum and vulva in pregnancy and the puerperium - delivered with postnatal complication	436766	Delivery record only (DELIV)	Delivery	Conditions	
Maternal distress - delivered	436183	Delivery record only (DELIV)	Delivery	Conditions	
Hyperemesis gravidarum with metabolic disturbance - delivered	436173	Delivery record only (DELIV)	Delivery	Conditions	
Retracted nipple in pregnancy, the puerperium or lactation - delivered	435612	Delivery record only (DELIV)	Delivery	Conditions	
Varicose veins of perineum and vulva in pregnancy and the puerperium - delivered	435610	Delivery record only (DELIV)	Delivery	Conditions	
Vasa previa - delivered	435609	Delivery record only (DELIV)	Delivery	Conditions	
Cervical incompetence - delivered with postnatal complication	435607	Delivery record only (DELIV)	Delivery	Conditions	
Varicose veins of legs in pregnancy and the puerperium - delivered with postnatal complication	435330	Delivery record only (DELIV)	Delivery	Conditions	
Postnatal deep vein thrombosis - delivered with postnatal complication	435031	Delivery record only (DELIV)	Delivery	Conditions	
Short cord - delivered	435020	Delivery record only (DELIV)	Delivery	Conditions	
Cracked nipple in pregnancy, the puerperium or lactation - delivered	434715	Delivery record only (DELIV)	Delivery	Conditions	
Puerperal pyrexia of unknown origin - delivered with postnatal complication	434713	Delivery record only (DELIV)	Delivery	Conditions	
Prolonged second stage - delivered	434431	Delivery record only (DELIV)	Delivery	Conditions	
Polyhydramnios - delivered	434427	Delivery record only (DELIV)	Delivery	Conditions	
Obstetric blood-clot pulmonary embolism - delivered with postnatal complication	434112	Delivery record only (DELIV)	Delivery	Conditions	
Tuberculosis in pregnancy, childbirth and the puerperium - delivered	433812	Delivery record only (DELIV)	Delivery	Conditions	

Retracted nipple in pregnancy, the puerperium or lactation - delivered with postnatal complication	433276	Delivery record only (DELIV)	Delivery	Conditions	
Oligohydramnios - delivered	433274	Delivery record only (DELIV)	Delivery	Conditions	
Amniotic fluid pulmonary embolism - delivered with postnatal complication	432388	Delivery record only (DELIV)	Delivery	Conditions	
Vascular lesions of cord - delivered	321367	Delivery record only (DELIV)	Delivery	Conditions	
Benign essential hypertension complicating pregnancy, childbirth and the puerperium - delivered with postnatal complication	320456	Delivery record only (DELIV)	Delivery	Conditions	
Benign essential hypertension complicating pregnancy, childbirth and the puerperium - delivered	314103	Delivery record only (DELIV)	Delivery	Conditions	
Maternal hypotension syndrome - delivered with postnatal problem	313829	Delivery record only (DELIV)	Delivery	Conditions	
Renal hypertension complicating pregnancy, childbirth and the puerperium - delivered with postnatal complication	201912	Delivery record only (DELIV)	Delivery	Conditions	
Obstetric pelvic hematoma - delivered	201368	Delivery record only (DELIV)	Delivery	Conditions	
Placenta previa without hemorrhage - delivered	201359	Delivery record only (DELIV)	Delivery	Conditions	
Premature rupture of membranes - delivered	200160	Delivery record only (DELIV)	Delivery	Conditions	
Renal hypertension complicating pregnancy, childbirth and the puerperium - delivered	200157	Delivery record only (DELIV)	Delivery	Conditions	
Obstructed labor caused by bony pelvis - delivered	199087	Delivery record only (DELIV)	Delivery	Conditions	
Rupture of uterus before labor - delivered	198816	Delivery record only (DELIV)	Delivery	Conditions	
Second degree perineal tear during delivery - delivered	198499	Delivery record only (DELIV)	Delivery	Conditions	
Disproportion - major pelvic abnormality - delivered	198486	Delivery record only (DELIV)	Delivery	Conditions	
Retroverted incarcerated gravid uterus - delivered	198216	Delivery record only (DELIV)	Delivery	Conditions	
Retroverted incarcerated gravid uterus - delivered with postnatal complication	197616	Delivery record only (DELIV)	Delivery	Conditions	
Obstetric inversion of uterus - delivered with postnatal problem	197048	Delivery record only (DELIV)	Delivery	Conditions	
Peripheral neuritis in pregnancy - delivered	197042	Delivery record only (DELIV)	Delivery	Conditions	
Primary uterine inertia - delivered	196762	Delivery record only (DELIV)	Delivery	Conditions	
Obstructed labor caused by pelvic soft tissues - delivered	196182	Delivery record only (DELIV)	Delivery	Conditions	
Liver disorder in pregnancy - delivered	196170	Delivery record only (DELIV)	Delivery	Conditions	
Rhesus isoimmunization - delivered	195878	Delivery record only (DELIV)	Delivery	Conditions	
Obstetric shock - delivered with postnatal problem	195025	Delivery record only (DELIV)	Delivery	Conditions	
Obstructed labor due to fetal malposition - delivered	194711	Delivery record only (DELIV)	Delivery	Conditions	
Fourth degree perineal tear during delivery - delivered	194429	Delivery record only (DELIV)	Delivery	Conditions	
Cesarean wound disruption - delivered with postnatal complication	194113	Delivery record only (DELIV)	Delivery	Conditions	
Placental abruption - delivered	193539	Delivery record only (DELIV)	Delivery	Conditions	
Fetal-maternal hemorrhage - delivered	193535	Delivery record only (DELIV)	Delivery	Conditions	
Third degree perineal tear during delivery - delivered	193275	Delivery record only (DELIV)	Delivery	Conditions	
Obstetric pelvic hematoma - delivered with postnatal problem	193271	Delivery record only (DELIV)	Delivery	Conditions	
Obstetric high vaginal laceration - delivered	193269	Delivery record only (DELIV)	Delivery	Conditions	
Placenta previa with hemorrhage - delivered	193264	Delivery record only (DELIV)	Delivery	Conditions	
First degree perineal tear during delivery - delivered	192978	Delivery record only (DELIV)	Delivery	Conditions	
Pelvic soft tissue abnormality in pregnancy, childbirth and the puerperium - delivered with postnatal complication	192971	Delivery record only (DELIV)	Delivery	Conditions	
Rupture of uterus during and after labor - delivered	192699	Delivery record only (DELIV)	Delivery	Conditions	
Obstetric perineal wound disruption - delivered with postnatal complication	192387	Delivery record only (DELIV)	Delivery	Conditions	
Secondary uterine inertia - delivered	192384	Delivery record only (DELIV)	Delivery	Conditions	
Transient hypertension of pregnancy - delivered with postnatal complication	141639	Delivery record only (DELIV)	Delivery	Conditions	
Eclampsia - delivered with postnatal complication	138811	Delivery record only (DELIV)	Delivery	Conditions	
Transient hypertension of pregnancy - delivered	137940	Delivery record only (DELIV)	Delivery	Conditions	
Pre-eclampsia or eclampsia with pre-existing hypertension - delivered	135601	Delivery record only (DELIV)	Delivery	Conditions	
Pre-eclampsia or eclampsia with pre-existing hypertension - delivered with postnatal complication	134414	Delivery record only (DELIV)	Delivery	Conditions	
Eclampsia - delivered	133816	Delivery record only (DELIV)	Delivery	Conditions	
Genitourinary tract infection in pregnancy - delivered	81357	Delivery record only (DELIV)	Delivery	Conditions	
Breast engorgement in pregnancy, the puerperium or lactation - delivered with postnatal complication	81086	Delivery record only (DELIV)	Delivery	Conditions	
Large fetus causing disproportion - delivered	80782	Delivery record only (DELIV)	Delivery	Conditions	
Genitourinary tract infection in pregnancy - delivered with postnatal complication	80778	Delivery record only (DELIV)	Delivery	Conditions	
Failure of lactation - delivered with postnatal complication	80479	Delivery record only (DELIV)	Delivery	Conditions	
Shoulder dystocia - delivered	80474	Delivery record only (DELIV)	Delivery	Conditions	
Obstetric non-purulent mastitis - delivered with postnatal complication	79897	Delivery record only (DELIV)	Delivery	Conditions	
Unstable lie - delivered	79889	Delivery record only (DELIV)	Delivery	Conditions	
Obstetric breast abscess - delivered with postnatal complication	78494	Delivery record only (DELIV)	Delivery	Conditions	
Breast engorgement in pregnancy, the puerperium or lactation - delivered	77624	Delivery record only (DELIV)	Delivery	Conditions	
Inlet pelvic contraction - delivered	77621	Delivery record only (DELIV)	Delivery	Conditions	
High head at term - delivered	77615	Delivery record only (DELIV)	Delivery	Conditions	
Suppressed lactation - delivered	77347	Delivery record only (DELIV)	Delivery	Conditions	
Failure of lactation - delivered	77056	Delivery record only (DELIV)	Delivery	Conditions	
Generally contracted pelvis - delivered	77052	Delivery record only (DELIV)	Delivery	Conditions	
Suppressed lactation - delivered with postnatal complication	76769	Delivery record only (DELIV)	Delivery	Conditions	
Asymptomatic bacteriuria in pregnancy - delivered	76761	Delivery record only (DELIV)	Delivery	Conditions	
Deep transverse arrest - delivered	75882	Delivery record only (DELIV)	Delivery	Conditions	
Obstetric damage to pelvic joints and ligaments - delivered	75326	Delivery record only (DELIV)	Delivery	Conditions	
Galactorrhea in pregnancy and the puerperium - delivered with postnatal complication	74717	Delivery record only (DELIV)	Delivery	Conditions	
Galactorrhea in pregnancy and the puerperium - delivered	74440	Delivery record only (DELIV)	Delivery	Conditions	

Obstetric breast abscess - delivered	74433	Delivery record only (DELIV)	Delivery	Conditions	
Outlet pelvic contraction - delivered	73824	Delivery record only (DELIV)	Delivery	Conditions	
Hydrocephalic disproportion - delivered	73538	Delivery record only (DELIV)	Delivery	Conditions	
Asymptomatic bacteriuria in pregnancy - delivered with postnatal complication	73526	Delivery record only (DELIV)	Delivery	Conditions	
Mixed feto-pelvic disproportion - delivered	72970	Delivery record only (DELIV)	Delivery	Conditions	
Fetus with chromosomal abnormality - delivered	72697	Delivery record only (DELIV)	Delivery	Conditions	
Prolapsed arm - delivered	72692	Delivery record only (DELIV)	Delivery	Conditions	
Delivery of Products of Conception, External Approach	2784578	Delivery record only (DELIV)	Delivery	Conditions	
Manual Extraction of Products of Conception, Retained, Via Natural or Artificial Opening Endoscopic	43018345	Delivery record only (DELIV)	Delivery	Conditions	
Manual Extraction of Products of Conception, Retained, Via Natural or Artificial Opening	43018344	Delivery record only (DELIV)	Delivery	Conditions	
Extraction of Products of Conception, Retained, Via Natural or Artificial Opening Endoscopic	2784574	Delivery record only (DELIV)	Delivery	Conditions	
Extraction of Products of Conception, Retained, Via Natural or Artificial Opening	2784573	Delivery record only (DELIV)	Delivery	Conditions	
Extraction of Products of Conception, Other, Via Natural or Artificial Opening	2784572	Delivery record only (DELIV)	Delivery	Conditions	
Extraction of Products of Conception, Internal Version, Via Natural or Artificial Opening	2784571	Delivery record only (DELIV)	Delivery	Conditions	
Extraction of Products of Conception, Vacuum, Via Natural or Artificial Opening	2784570	Delivery record only (DELIV)	Delivery	Conditions	
Extraction of Products of Conception, High Forceps, Via Natural or Artificial Opening	2784569	Delivery record only (DELIV)	Delivery	Conditions	
Extraction of Products of Conception, Mid Forceps, Via Natural or Artificial Opening	2784568	Delivery record only (DELIV)	Delivery	Conditions	
Extraction of Products of Conception, Low Forceps, Via Natural or Artificial Opening	2784567	Delivery record only (DELIV)	Delivery	Conditions	
Extraction of Products of Conception, Extraperitoneal, Open Approach	2784566	Delivery record only (DELIV)	Delivery	Conditions	
Extraction of Products of Conception, Low, Open Approach	2784565	Delivery record only (DELIV)	Delivery	Conditions	
Extraction of Products of Conception, High, Open Approach	2784564	Delivery record only (DELIV)	Delivery	Conditions	
Premature 28 week quadruplet	3174212	Livebirth (LB)	Delivery	Conditions	
Premature birth of newborn sextuplets	4008884	Livebirth (LB)	Delivery	Conditions	
Single live birth	4014295	Livebirth (LB)	Delivery	Conditions	
Twins - both live born	4014296	Livebirth (LB)	Delivery	Conditions	
Twins - one still and one live born	4014455	Livebirth (LB)	Delivery	Conditions	
Triplets - all live born	4014456	Livebirth (LB)	Delivery	Conditions	
Triplets - two live and one stillborn	4015421	Livebirth (LB)	Delivery	Conditions	
Triplets - one live and two stillborn	4015422	Livebirth (LB)	Delivery	Conditions	
Premature birth of newborn twins	4029786	Livebirth (LB)	Delivery	Conditions	
Term birth of identical twins, both living	4049333	Livebirth (LB)	Delivery	Conditions	
Term birth of newborn male	4052512	Livebirth (LB)	Delivery	Conditions	
Term birth of newborn	4054968	Livebirth (LB)	Delivery	Conditions	
Term birth of multiple newborns	4066292	Livebirth (LB)	Delivery	Conditions	
Premature birth of multiple newborns	4069200	Livebirth (LB)	Delivery	Conditions	
Premature birth of newborn quintuplets	4082863	Livebirth (LB)	Delivery	Conditions	
Livebirth	4092289	Livebirth (LB)	Delivery	Conditions	
Triplet birth	4094046	Livebirth (LB)	Delivery	Conditions	
Premature birth of fraternal twins, both living	4097427	Livebirth (LB)	Delivery	Conditions	
Twin birth	4101844	Livebirth (LB)	Delivery	Conditions	
Premature birth of newborn triplets	4107401	Livebirth (LB)	Delivery	Conditions	
Premature birth of identical twins, both living	4142021	Livebirth (LB)	Delivery	Conditions	
Premature birth of newborn quadruplets	4145125	Livebirth (LB)	Delivery	Conditions	
Premature birth of fraternal twins, one living, one stillborn	4147043	Livebirth (LB)	Delivery	Conditions	
Multiple birth	4163851	Livebirth (LB)	Delivery	Conditions	
Premature birth of newborn male	4172135	Livebirth (LB)	Delivery	Conditions	
Term birth of newborn twins	4178275	Livebirth (LB)	Delivery	Conditions	
Term birth of newborn quadruplets	4193224	Livebirth (LB)	Delivery	Conditions	
Term birth of newborn sextuplets	4199146	Livebirth (LB)	Delivery	Conditions	
Term birth of fraternal twins, both living	4227728	Livebirth (LB)	Delivery	Conditions	
Premature birth of newborn female	4241228	Livebirth (LB)	Delivery	Conditions	
Term birth of identical twins, one living, one stillborn	4243026	Livebirth (LB)	Delivery	Conditions	
Term birth of newborn female	4246654	Livebirth (LB)	Delivery	Conditions	
Premature birth of newborn	4272248	Livebirth (LB)	Delivery	Conditions	
Term birth of newborn triplets	4307237	Livebirth (LB)	Delivery	Conditions	
Premature birth of identical twins, one living, one stillborn	4310910	Livebirth (LB)	Delivery	Conditions	
Term birth of fraternal twins, one living, one stillborn	4330570	Livebirth (LB)	Delivery	Conditions	
Term birth of newborn quintuplets	4336090	Livebirth (LB)	Delivery	Conditions	
Twin live born in hospital by vaginal delivery	42535052	Livebirth (LB)	Delivery	Conditions	
Triplets, some live born	45757165	Livebirth (LB)	Delivery	Conditions	
Quadruplets, all live born	45757166	Livebirth (LB)	Delivery	Conditions	
Quintuplets, all live born	45757167	Livebirth (LB)	Delivery	Conditions	
Quintuplets, some live born	45757168	Livebirth (LB)	Delivery	Conditions	
Sextuplets, some live born	45757169	Livebirth (LB)	Delivery	Conditions	
Quadruplet birth	45765500	Livebirth (LB)	Delivery	Conditions	
Quintuplet birth	45765501	Livebirth (LB)	Delivery	Conditions	
Sextuplet birth	45765502	Livebirth (LB)	Delivery	Conditions	
Sextuplets, all live born	45772082	Livebirth (LB)	Delivery	Conditions	
Quadruplets, some live born	45773428	Livebirth (LB)	Delivery	Conditions	
Multiple liveborn in hospital by vaginal delivery	42539267	Livebirth (LB)	Delivery	Conditions	

Triplet liveborn in hospital by cesarean section	42539210	Livebirth (LB)	Delivery	Conditions	
Single liveborn born in hospital by cesarean section	40483521	Livebirth (LB)	Delivery	Conditions	
Liveborn born in hospital by cesarean section	40483126	Livebirth (LB)	Delivery	Conditions	
Twin liveborn born in hospital by cesarean section	40483101	Livebirth (LB)	Delivery	Conditions	
Twin liveborn born in hospital	40483084	Livebirth (LB)	Delivery	Conditions	
Liveborn born in hospital	40482735	Livebirth (LB)	Delivery	Conditions	
Twin liveborn born outside hospital	36717637	Livebirth (LB)	Delivery	Conditions	
Multiple liveborn other than twins born outside hospital	36713469	Livebirth (LB)	Delivery	Conditions	
Multiple liveborn other than twins born in hospital	36713468	Livebirth (LB)	Delivery	Conditions	
Singleton liveborn unspecified as to place of birth	36713467	Livebirth (LB)	Delivery	Conditions	
Singleton liveborn born outside hospital	36713466	Livebirth (LB)	Delivery	Conditions	
Singleton liveborn born in hospital	36713465	Livebirth (LB)	Delivery	Conditions	
Single liveborn born in hospital by vaginal delivery	36713074	Livebirth (LB)	Delivery	Conditions	
Are you currently pregnant?	1585811	Pregnancy	Pregnancy	Survey	
Pregnant	4299535	Pregnancy	Pregnancy	Conditions	
High risk pregnancy	4188598	Pregnancy	Pregnancy	Conditions	
High risk pregnancy due to history of preterm labor	40480278	Pregnancy	Pregnancy	Conditions	
Intrauterine pregnancy	197031	Pregnancy	Pregnancy	Conditions	
Unplanned pregnancy	4307820	Pregnancy	Pregnancy	Conditions	
Normal pregnancy in primigravida	36684864	Pregnancy	Pregnancy	Conditions	
Combined tubal and intrauterine pregnancy	40483581	Pregnancy	Pregnancy	Conditions	
Pregnant - on history	4061686	Pregnancy	Pregnancy	Conditions	
Surrogate pregnancy	4012559	Pregnancy	Pregnancy	Conditions	
Pregnancy unplanned but wanted	4060237	Pregnancy	Pregnancy	Conditions	
High risk pregnancy due to recurrent pregnancy loss	37018878	Pregnancy	Pregnancy	Conditions	
Normal pregnancy	4217975	Pregnancy	Pregnancy	Conditions	
Unwanted pregnancy	4239301	Pregnancy	Pregnancy	Conditions	
Pregnant - urine test confirms	4059981	Pregnancy	Pregnancy	Conditions	
24 HR paroxetine hydrochloride 37.5 MG Extended Release Oral Tablet [Paxil]	35604762	Antidepressant	Depression	Drugs	
venlafaxine 100 MG Oral Tablet [Effexor]	19039817	Antidepressant	Depression	Drugs	
imipramine	778268	Antidepressant	Depression	Drugs	
vilazodone	40234834	Antidepressant	Depression	Drugs	
doxepin hydrochloride 150 MG Oral Capsule	40224891	Antidepressant	Depression	Drugs	
nortriptyline 50 MG Oral Capsule	19019413	Antidepressant	Depression	Drugs	
clomipramine hydrochloride 50 MG Oral Capsule	40163070	Antidepressant	Depression	Drugs	
fluoxetine 10 MG Oral Tablet	19079985	Antidepressant	Depression	Drugs	
escitalopram 20 MG Oral Tablet [Lexapro]	715966	Antidepressant	Depression	Drugs	
amitriptyline hydrochloride 100 MG Oral Tablet	40162683	Antidepressant	Depression	Drugs	
citalopram 20 MG Oral Tablet [Citalopraam]	19113503	Antidepressant	Depression	Drugs	
trazodone hydrochloride 50 MG Oral Tablet [Desyrel]	40163474	Antidepressant	Depression	Drugs	
mirtazapine 45 MG Disintegrating Oral Tablet	725177	Antidepressant	Depression	Drugs	
mirtazapine 15 MG Disintegrating Oral Tablet	19070747	Antidepressant	Depression	Drugs	
doxepin	738156	Antidepressant	Depression	Drugs	
escitalopram 5 MG Oral Tablet	715962	Antidepressant	Depression	Drugs	
venlafaxine 100 MG Oral Tablet	19079835	Antidepressant	Depression	Drugs	
escitalopram 10 MG Oral Tablet [Lexapro]	715965	Antidepressant	Depression	Drugs	
12 HR bupropion hydrochloride 200 MG Extended Release Oral Tablet	40221864	Antidepressant	Depression	Drugs	
milnacipran	19080226	Antidepressant	Depression	Drugs	
24 HR venlafaxine 150 MG Extended Release Oral Capsule [Effexor]	743794	Antidepressant	Depression	Drugs	
desipramine hydrochloride 100 MG Oral Tablet	40237422	Antidepressant	Depression	Drugs	
citalopram 40 MG Oral Tablet [Cipramil]	19054100	Antidepressant	Depression	Drugs	
venlafaxine 37.5 MG Oral Tablet [Effexor]	19039819	Antidepressant	Depression	Drugs	
doxepin hydrochloride 10 MG Oral Capsule	40224875	Antidepressant	Depression	Drugs	
amitriptyline hydrochloride 10 MG Oral Tablet [Elavil]	40162673	Antidepressant	Depression	Drugs	
24 HR venlafaxine 225 MG Extended Release Oral Tablet	19132561	Antidepressant	Depression	Drugs	
venlafaxine 75 MG Oral Tablet	743752	Antidepressant	Depression	Drugs	
24 HR desvenlafaxine succinate 100 MG Extended Release Oral Tablet [Pristiq]	19129666	Antidepressant	Depression	Drugs	
Smoking Cessation 12 HR bupropion hydrochloride 150 MG Extended Release Oral Tablet [Zyban]	40221863	Antidepressant	Depression	Drugs	
24 HR venlafaxine 75 MG Extended Release Oral Capsule [Effexor]	743793	Antidepressant	Depression	Drugs	
fluoxetine 40 MG Oral Capsule [Prozac]	755735	Antidepressant	Depression	Drugs	
duloxetine 20 MG Delayed Release Oral Capsule [Cymbalta]	715295	Antidepressant	Depression	Drugs	
24 HR bupropion hydrochloride 300 MG Extended Release Oral Tablet [Wellbutrin]	40221876	Antidepressant	Depression	Drugs	
duloxetine 30 MG Delayed Release Oral Capsule [Cymbalta]	715298	Antidepressant	Depression	Drugs	
vortioxetine 20 MG Oral Tablet	44506612	Antidepressant	Depression	Drugs	
fluvoxamine maleate 100 MG Oral Tablet	40174725	Antidepressant	Depression	Drugs	
nortriptyline 75 MG Oral Capsule	19019414	Antidepressant	Depression	Drugs	
venlafaxine 37.5 MG Oral Tablet	743720	Antidepressant	Depression	Drugs	
doxepin hydrochloride 25 MG Oral Capsule	40224897	Antidepressant	Depression	Drugs	
fluoxetine 20 MG Oral Capsule [Selfemra]	19130466	Antidepressant	Depression	Drugs	
desipramine hydrochloride 50 MG Oral Tablet	40237441	Antidepressant	Depression	Drugs	
bupropion hydrochloride 75 MG Oral Tablet	40222092	Antidepressant	Depression	Drugs	

trazodone hydrochloride 300 MG Oral Tablet	40163468	Antidepressant	Depression	Drugs	
sertraline 25 MG Oral Tablet [Zoloft]	739202	Antidepressant	Depression	Drugs	
24 HR paroxetine hydrochloride 12.5 MG Extended Release Oral Tablet	35604757	Antidepressant	Depression	Drugs	
citalopram 10 MG Oral Tablet [Celexa]	797633	Antidepressant	Depression	Drugs	
24 HR venlafaxine 150 MG Extended Release Oral Tablet	19132560	Antidepressant	Depression	Drugs	
24 HR bupropion hydrochloride 150 MG Extended Release Oral Tablet [Wellbutrin]	40221873	Antidepressant	Depression	Drugs	
bupropion hydrochloride 100 MG Oral Tablet	40222060	Antidepressant	Depression	Drugs	
mirtazapine 45 MG Oral Tablet	725179	Antidepressant	Depression	Drugs	
paroxetine hydrochloride 30 MG Oral Tablet	35604581	Antidepressant	Depression	Drugs	
fluvoxamine maleate 25 MG Oral Tablet	40174735	Antidepressant	Depression	Drugs	
milnacipran hydrochloride 50 MG Oral Tablet [Savella]	19080256	Antidepressant	Depression	Drugs	
24 HR desvenlafaxine succinate 100 MG Extended Release Oral Tablet	1593107	Antidepressant	Depression	Drugs	
fluoxetine 4 MG/ML Oral Solution	19077464	Antidepressant	Depression	Drugs	
doxepin hydrochloride 10 MG Oral Capsule [Sinequan]	40224877	Antidepressant	Depression	Drugs	
trazodone hydrochloride 100 MG Oral Tablet [Desyrel]	40163461	Antidepressant	Depression	Drugs	
fluvoxamine maleate 100 MG Oral Tablet [Luvox]	40174726	Antidepressant	Depression	Drugs	
citalopram 20 MG Oral Tablet [Celexa]	797619	Antidepressant	Depression	Drugs	
fluoxetine 20 MG Oral Tablet	755700	Antidepressant	Depression	Drugs	
venlafaxine 25 MG Oral Tablet	743718	Antidepressant	Depression	Drugs	
alanine 20.7 MG/ML / arginine 11.5 MG/ML / glycine 10.3 MG/ML / histidine 4.8 MG/ML / isoleucine 6 MG/ML / leucine 7.3 MG/ML / lysine 5.8 MG/ML / methionine 4 MG/ML / phenylalanine 5.6 MG/ML / proline 6.8 MG/ML / serine 5 MG/ML / threonine 4.2 MG/ML /...	19131343	Antidepressant	Depression	Drugs	
vortioxetine 10 MG Oral Tablet [Trintellix]	42629569	Antidepressant	Depression	Drugs	
trazodone hydrochloride 150 MG Oral Tablet [Desyrel]	40163465	Antidepressant	Depression	Drugs	
vilazodone hydrochloride 40 MG Oral Tablet [Viibryd]	40234847	Antidepressant	Depression	Drugs	
vortioxetine 20 MG Oral Tablet [Trintellix]	42629573	Antidepressant	Depression	Drugs	
doxepin hydrochloride 100 MG Oral Capsule	40224885	Antidepressant	Depression	Drugs	
12 HR bupropion hydrochloride 100 MG Extended Release Oral Tablet [Wellbutrin]	40221858	Antidepressant	Depression	Drugs	
fluvoxamine	751412	Antidepressant	Depression	Drugs	
vilazodone hydrochloride 20 MG Oral Tablet [Viibryd]	40234843	Antidepressant	Depression	Drugs	
amitriptyline hydrochloride 25 MG Oral Tablet [Endep]	40162719	Antidepressant	Depression	Drugs	
doxepin hydrochloride 50 MG Oral Capsule	40224903	Antidepressant	Depression	Drugs	
desipramine hydrochloride 10 MG Oral Tablet	40237418	Antidepressant	Depression	Drugs	
imipramine hydrochloride 25 MG Oral Tablet	778296	Antidepressant	Depression	Drugs	
tranylcypromine 10 MG Oral Tablet [Parnate]	19004820	Antidepressant	Depression	Drugs	
24 HR venlafaxine 37.5 MG Extended Release Oral Capsule [Effexor]	743795	Antidepressant	Depression	Drugs	
venlafaxine 50 MG Oral Tablet	743753	Antidepressant	Depression	Drugs	
{7 (vilazodone hydrochloride 10 MG Oral Tablet [Viibryd]) / 7 (vilazodone hydrochloride 20 MG Oral Tablet [Viibryd]) / 16 (vilazodone hydrochloride 40 MG Oral Tablet [Viibryd]) } Pack [Viibryd 10/20/40 30 Day Pack]	40235110	Antidepressant	Depression	Drugs	
escitalopram 5 MG Oral Tablet [Lexapro]	19102734	Antidepressant	Depression	Drugs	
phenelzine 15 MG Oral Tablet	733898	Antidepressant	Depression	Drugs	
12 HR bupropion hydrochloride 150 MG Extended Release Oral Tablet [Budeprion]	40221860	Antidepressant	Depression	Drugs	
nortriptyline 10 MG Oral Capsule [Aventyl]	40239274	Antidepressant	Depression	Drugs	
amitriptyline hydrochloride 75 MG Oral Tablet	40162737	Antidepressant	Depression	Drugs	
clomipramine hydrochloride 25 MG Oral Capsule	40163063	Antidepressant	Depression	Drugs	
trimipramine 100 MG Oral Capsule	705794	Antidepressant	Depression	Drugs	
mirtazapine 7.5 MG Oral Tablet	19112586	Antidepressant	Depression	Drugs	
doxepin hydrochloride 75 MG Oral Capsule	40224915	Antidepressant	Depression	Drugs	
mirtazapine 15 MG Oral Tablet [Remeron]	725172	Antidepressant	Depression	Drugs	
protriptyline hydrochloride 10 MG Oral Tablet	40175376	Antidepressant	Depression	Drugs	
paroxetine hydrochloride 10 MG Oral Tablet	35604569	Antidepressant	Depression	Drugs	
paroxetine mesylate 40 MG Oral Tablet [Pexeva]	722153	Antidepressant	Depression	Drugs	
citalopram 40 MG Oral Tablet [Celexa]	797620	Antidepressant	Depression	Drugs	
mirtazapine 30 MG Oral Tablet [Remeron]	725173	Antidepressant	Depression	Drugs	
vilazodone hydrochloride 10 MG Oral Tablet [Viibryd]	40234839	Antidepressant	Depression	Drugs	
12 HR bupropion hydrochloride 200 MG Extended Release Oral Tablet [Wellbutrin]	40221866	Antidepressant	Depression	Drugs	
nortriptyline 50 MG Oral Capsule [Pamelor]	19041194	Antidepressant	Depression	Drugs	
desvenlafaxine	717607	Antidepressant	Depression	Drugs	
amitriptyline hydrochloride 50 MG Oral Tablet [Vanatrip]	40162732	Antidepressant	Depression	Drugs	
clomipramine	798834	Antidepressant	Depression	Drugs	
doxepin hydrochloride 25 MG Oral Capsule [Sinequan]	40224899	Antidepressant	Depression	Drugs	
nefazodone hydrochloride 100 MG Oral Tablet	40238415	Antidepressant	Depression	Drugs	
fluvoxamine maleate 50 MG Oral Tablet	40174739	Antidepressant	Depression	Drugs	
desipramine	716968	Antidepressant	Depression	Drugs	
nefazodone	714684	Antidepressant	Depression	Drugs	
24 HR paroxetine hydrochloride 25 MG Extended Release Oral Tablet	35604759	Antidepressant	Depression	Drugs	
fluoxetine 10 MG Oral Capsule [Prozac]	19029479	Antidepressant	Depression	Drugs	
12 HR bupropion hydrochloride 90 MG / naltrexone hydrochloride 8 MG Extended Release Oral Tablet [Contrave]	45774490	Antidepressant	Depression	Drugs	
duloxetine 40 MG Delayed Release Oral Capsule [Irenka]	46233914	Antidepressant	Depression	Drugs	
nefazodone hydrochloride 150 MG Oral Tablet	40238419	Antidepressant	Depression	Drugs	

paroxetine hydrochloride 25 MG Extended Release Oral Tablet [Paxil]	722159	Antidepressant	Depression	Drugs	
Smoking Cessation 12 HR bupropion hydrochloride 150 MG Extended Release Oral Tablet	36249641	Antidepressant	Depression	Drugs	
paroxetine mesylate 30 MG Oral Tablet [Pexeva]	722152	Antidepressant	Depression	Drugs	
24 HR paroxetine hydrochloride 12.5 MG Extended Release Oral Tablet [Paxil]	35604758	Antidepressant	Depression	Drugs	
paroxetine mesylate 20 MG Oral Tablet [Pexeva]	722121	Antidepressant	Depression	Drugs	
desipramine hydrochloride 25 MG Oral Tablet	40237432	Antidepressant	Depression	Drugs	
imipramine hydrochloride 50 MG Oral Tablet	778299	Antidepressant	Depression	Drugs	
24 HR venlafaxine 75 MG Extended Release Oral Tablet	19132563	Antidepressant	Depression	Drugs	
24 HR desvenlafaxine succinate 50 MG Extended Release Oral Tablet [Pristiq]	19129683	Antidepressant	Depression	Drugs	
fluoxetine 60 MG Oral Tablet	40243919	Antidepressant	Depression	Drugs	
nefazodone hydrochloride 200 MG Oral Tablet	40238423	Antidepressant	Depression	Drugs	
paroxetine hydrochloride 20 MG Oral Tablet [Paxil]	19035312	Antidepressant	Depression	Drugs	
fluvoxamine maleate 50 MG Oral Tablet [Luvox]	40174740	Antidepressant	Depression	Drugs	
protriptyline hydrochloride 5 MG Oral Tablet	40175380	Antidepressant	Depression	Drugs	
fluoxetine 20 MG Oral Capsule [Prozac]	19004895	Antidepressant	Depression	Drugs	
vortioxetine 10 MG Oral Tablet	44506611	Antidepressant	Depression	Drugs	
tranylcypromine 10 MG Oral Tablet	703544	Antidepressant	Depression	Drugs	
nortriptyline 25 MG Oral Capsule [Aventyl]	40239277	Antidepressant	Depression	Drugs	
sertraline 20 MG/ML Oral Solution	40165282	Antidepressant	Depression	Drugs	
fluoxetine 10 MG Oral Capsule [Selfemra]	19130449	Antidepressant	Depression	Drugs	
12 HR bupropion hydrochloride 150 MG Extended Release Oral Tablet [Wellbutrin]	40221862	Antidepressant	Depression	Drugs	
phenelzine	733896	Antidepressant	Depression	Drugs	
desipramine hydrochloride 75 MG Oral Tablet	40237445	Antidepressant	Depression	Drugs	
24 HR venlafaxine 37.5 MG Extended Release Oral Tablet	19132562	Antidepressant	Depression	Drugs	
PMDD fluoxetine 20 MG Oral Tablet [Sarafem]	755854	Antidepressant	Depression	Drugs	
24 HR desvenlafaxine succinate 50 MG Extended Release Oral Tablet	1593112	Antidepressant	Depression	Drugs	
24 HR desvenlafaxine 100 MG Extended Release Oral Tablet [Khedezla]	43559999	Antidepressant	Depression	Drugs	
escitalopram 1 MG/ML Oral Solution	715964	Antidepressant	Depression	Drugs	
mirtazapine 15 MG Disintegrating Oral Tablet [Remeron]	725206	Antidepressant	Depression	Drugs	
amitriptyline hydrochloride 150 MG Oral Tablet	40162695	Antidepressant	Depression	Drugs	
alanine 12.8 MG/ML / arginine 9.8 MG/ML / glycine 12.8 MG/ML / histidine 3 MG/ML / isoleucine 7.2 MG/ML / leucine 9.4 MG/ML / lysine 7.2 MG/ML / methionine 4 MG/ML / phenylalanine 4.4 MG/ML / proline 8.6 MG/ML / serine 4.2 MG/ML / threonine 5.2 MG/ML /...	19133483	Antidepressant	Depression	Drugs	
bupropion hydrochloride 75 MG Oral Tablet [Wellbutrin]	40222093	Antidepressant	Depression	Drugs	
doxepin hydrochloride 50 MG/ML Topical Cream	40224909	Antidepressant	Depression	Drugs	
amoxapine 25 MG Oral Tablet	713132	Antidepressant	Depression	Drugs	
24 HR desvenlafaxine succinate 25 MG Extended Release Oral Tablet	46221214	Antidepressant	Depression	Drugs	
bupropion hydrochloride 100 MG Oral Tablet [Wellbutrin]	40222061	Antidepressant	Depression	Drugs	
doxepin hydrochloride 75 MG Oral Capsule [Sinequan]	40224917	Antidepressant	Depression	Drugs	
venlafaxine 75 MG Oral Tablet [Effexor]	19039822	Antidepressant	Depression	Drugs	
paroxetine hydrochloride 10 MG Oral Tablet [Paxil]	722064	Antidepressant	Depression	Drugs	
alanine 10.4 MG/ML / arginine 5.75 MG/ML / calcium chloride 0.004 MEQ/ML / dibasic potassium phosphate 2.61 MG/ML / glucose 150 MG/ML / glycine 5.15 MG/ML / histidine 2.4 MG/ML / isoleucine 3 MG/ML / leucine 3.65 MG/ML / lysine 2.9 MG/ML / magnesium ch...	19131082	Antidepressant	Depression	Drugs	
maprotiline hydrochloride 25 MG Oral Tablet	42800984	Antidepressant	Depression	Drugs	
protriptyline hydrochloride 5 MG Oral Tablet [Vivactil]	40175381	Antidepressant	Depression	Drugs	
imipramine hydrochloride 25 MG Oral Tablet [Tofranil]	778297	Antidepressant	Depression	Drugs	
mirtazapine 45 MG Oral Tablet [Remeron]	725174	Antidepressant	Depression	Drugs	
24 HR desvenlafaxine 50 MG Extended Release Oral Tablet [Khedezla]	43560000	Antidepressant	Depression	Drugs	
imipramine hydrochloride 10 MG Oral Tablet [Tofranil]	778359	Antidepressant	Depression	Drugs	
imipramine hydrochloride 10 MG Oral Tablet	778357	Antidepressant	Depression	Drugs	
doxepin 3 MG Oral Tablet [Silenor]	40173385	Antidepressant	Depression	Drugs	
imipramine hydrochloride 50 MG Oral Tablet [Tofranil]	19134040	Antidepressant	Depression	Drugs	
doxepin hydrochloride 50 MG Oral Capsule [Sinequan]	40224905	Antidepressant	Depression	Drugs	
milnacipran hydrochloride 100 MG Oral Tablet [Savella]	19080251	Antidepressant	Depression	Drugs	
24 HR bupropion hydrochloride 150 MG Extended Release Oral Tablet [Budeprion]	40221872	Antidepressant	Depression	Drugs	
24 HR desvenlafaxine succinate 25 MG Extended Release Oral Tablet [Pristiq]	46221216	Antidepressant	Depression	Drugs	
paroxetine mesylate 10 MG Oral Tablet [Pexeva]	19113754	Antidepressant	Depression	Drugs	
24 HR fluvoxamine maleate 150 MG Extended Release Oral Capsule	40173466	Antidepressant	Depression	Drugs	
amitriptyline hydrochloride 75 MG Oral Tablet [Endep]	40162739	Antidepressant	Depression	Drugs	
24 HR desvenlafaxine 100 MG Extended Release Oral Tablet	19129664	Antidepressant	Depression	Drugs	
amitriptyline hydrochloride 100 MG Oral Tablet [Endep]	40162685	Antidepressant	Depression	Drugs	
citalopram 2 MG/ML Oral Solution	19075393	Antidepressant	Depression	Drugs	
doxepin hydrochloride 150 MG Oral Capsule [Sinequan]	40224893	Antidepressant	Depression	Drugs	
vortioxetine 5 MG Oral Tablet [Trintellix]	42629575	Antidepressant	Depression	Drugs	
duloxetine 40 MG Delayed Release Oral Capsule	19123437	Antidepressant	Depression	Drugs	
24 HR bupropion hydrochloride 450 MG Extended Release Oral Tablet [Forfivo]	42707260	Antidepressant	Depression	Drugs	
alanine 8.8 MG/ML / arginine 4.89 MG/ML / calcium chloride 0.004 MEQ/ML / dibasic potassium phosphate 2.61 MG/ML / glucose 50 MG/ML / glycine 4.38 MG/ML / histidine 2.04 MG/ML / isoleucine 2.55 MG/ML / leucine 3.11 MG/ML / lysine 2.47 MG/ML / magnesium...	19131065	Antidepressant	Depression	Drugs	

bupropion hydrochloride 150 MG Extended Release Oral Tablet	40222065	Antidepressant	Depression	Drugs	
PMDD fluoxetine 10 MG Oral Tablet [Sarafem]	755853	Antidepressant	Depression	Drugs	
paroxetine hydrochloride 12.5 MG Extended Release Oral Tablet [Paxil]	722158	Antidepressant	Depression	Drugs	
amitriptyline hydrochloride 25 MG / perphenazine 2 MG Oral Tablet	40162704	Antidepressant	Depression	Drugs	
doxepin hydrochloride 10 MG/ML Oral Solution	40224881	Antidepressant	Depression	Drugs	
doxepin 3 MG Oral Tablet	40173384	Antidepressant	Depression	Drugs	
paroxetine hydrochloride 37.5 MG Extended Release Oral Tablet [Paxil]	722160	Antidepressant	Depression	Drugs	
paroxetine hydrochloride 30 MG Oral Tablet [Paxil]	19035313	Antidepressant	Depression	Drugs	
bupropion hydrochloride 300 MG Extended Release Oral Tablet	40222084	Antidepressant	Depression	Drugs	
amitriptyline hydrochloride 50 MG Oral Tablet [Domical]	19025666	Antidepressant	Depression	Drugs	
24 HR desvenlafaxine 50 MG Extended Release Oral Tablet	19129681	Antidepressant	Depression	Drugs	
12 HR bupropion hydrochloride 90 MG / naltrexone hydrochloride 8 MG Extended Release Oral Tablet	45774486	Antidepressant	Depression	Drugs	
amitriptyline hydrochloride 10 MG / perphenazine 2 MG Oral Tablet	40162658	Antidepressant	Depression	Drugs	
nortriptyline 75 MG Oral Capsule [Pamelor]	19041328	Antidepressant	Depression	Drugs	
amitriptyline hydrochloride 25 MG Oral Tablet [Elavil]	40162718	Antidepressant	Depression	Drugs	
alanine 8.8 MG/ML / arginine 4.89 MG/ML / calcium chloride 0.004 MEQ/ML / dibasic potassium phosphate 2.61 MG/ML / glucose 100 MG/ML / glycine 4.38 MG/ML / histidine 2.04 MG/ML / isoleucine 2.55 MG/ML / leucine 3.11 MG/ML / lysine 2.47 MG/ML / magnesiu...	40235428	Antidepressant	Depression	Drugs	
{3 (0.2 ML) (esketamine 140 MG/ML Nasal Spray [Spravato]) } Pack [Spravato 84 MG Dose Kit]	1366735	Antidepressant	Depression	Drugs	
alanine 10.4 MG/ML / arginine 5.75 MG/ML / glucose 200 MG/ML / glycine 5.15 MG/ML / histidine 2.4 MG/ML / isoleucine 3 MG/ML / leucine 3.65 MG/ML / lysine 2.9 MG/ML / methionine 2 MG/ML / phenylalanine 2.8 MG/ML / proline 3.4 MG/ML / serine 2.5 MG/ML /...	19130815	Antidepressant	Depression	Drugs	
venlafaxine 25 MG Oral Tablet [Effexor]	19039818	Antidepressant	Depression	Drugs	
24 HR paroxetine hydrochloride 37.5 MG Extended Release Oral Tablet	35604761	Antidepressant	Depression	Drugs	
doxepin 6 MG Oral Tablet	40173388	Antidepressant	Depression	Drugs	
bupropion hydrochloride 100 MG Extended Release Oral Tablet	40222057	Antidepressant	Depression	Drugs	
amitriptyline hydrochloride 50 MG Oral Tablet [Endep]	40162731	Antidepressant	Depression	Drugs	
milnacipran hydrochloride 25 MG Oral Tablet [Savella]	19080258	Antidepressant	Depression	Drugs	
duloxetine hydrochloride 60 MG Extended Release Oral Capsule	715292	Antidepressant	Depression	Drugs	
mirtazapine 30 MG Disintegrating Oral Tablet	19070748	Antidepressant	Depression	Drugs	
paroxetine mesylate 7.5 MG Oral Capsule [Brisdelle]	43532895	Antidepressant	Depression	Drugs	
amoxapine	713109	Antidepressant	Depression	Drugs	
24 HR bupropion hydrochloride 450 MG Extended Release Oral Tablet	42707259	Antidepressant	Depression	Drugs	
12 HR bupropion hydrochloride 100 MG Extended Release Oral Tablet [Budeprion]	40221857	Antidepressant	Depression	Drugs	
nefazodone hydrochloride 150 MG Oral Tablet [Serzone]	40238420	Antidepressant	Depression	Drugs	
paroxetine hydrochloride 40 MG Oral Tablet [Paxil]	19046308	Antidepressant	Depression	Drugs	
paroxetine mesylate 7.5 MG Oral Capsule	43532894	Antidepressant	Depression	Drugs	
vortioxetine	44507700	Antidepressant	Depression	Drugs	
imipramine pamoate 75 MG Oral Capsule	778322	Antidepressant	Depression	Drugs	
nortriptyline 25 MG Oral Capsule [Pamelor]	19041175	Antidepressant	Depression	Drugs	
24 HR bupropion hydrochloride 300 MG Extended Release Oral Tablet [Budeprion]	40221875	Antidepressant	Depression	Drugs	
Sprinkle duloxetine 60 MG Delayed Release Oral Capsule	37496767	Antidepressant	Depression	Drugs	
doxepin 6 MG Oral Tablet [Silenor]	40173389	Antidepressant	Depression	Drugs	
amoxapine 50 MG Oral Tablet	713133	Antidepressant	Depression	Drugs	
24 HR bupropion hydrobromide 522 MG Extended Release Oral Tablet [Aplenzin]	40221882	Antidepressant	Depression	Drugs	
doxepin hydrochloride 100 MG Oral Capsule [Sinequan]	40224887	Antidepressant	Depression	Drugs	
trazodone hydrochloride 300 MG Oral Tablet [Desyrel]	40163469	Antidepressant	Depression	Drugs	
milnacipran hydrochloride 25 MG Oral Tablet	19080257	Antidepressant	Depression	Drugs	
{5 (milnacipran hydrochloride 12.5 MG Oral Tablet) / 8 (milnacipran hydrochloride 25 MG Oral Tablet) / 42 (milnacipran hydrochloride 50 MG Oral Tablet) } Pack	19133788	Antidepressant	Depression	Drugs	
vortioxetine 5 MG Oral Tablet	44506613	Antidepressant	Depression	Drugs	
amitriptyline hydrochloride 25 MG Oral Tablet [Domical]	19025664	Antidepressant	Depression	Drugs	
clomipramine hydrochloride 75 MG Oral Capsule	40163076	Antidepressant	Depression	Drugs	
PMDD fluoxetine 20 MG Oral Tablet	740118	Antidepressant	Depression	Drugs	
24 HR paroxetine hydrochloride 25 MG Extended Release Oral Tablet [Paxil]	35604760	Antidepressant	Depression	Drugs	
phenelzine 15 MG Oral Tablet [Nardil]	19004818	Antidepressant	Depression	Drugs	
alanine 21.7 MG/ML / arginine 14.7 MG/ML / aspartate 4.34 MG/ML / glutamate 7.49 MG/ML / glycine 10.4 MG/ML / histidine 8.94 MG/ML / isoleucine 7.49 MG/ML / leucine 10.4 MG/ML / lysine 11.8 MG/ML / methionine 7.49 MG/ML / phenylalanine 10.4 MG/ML / pro...	19130883	Antidepressant	Depression	Drugs	
bupropion hydrochloride 300 MG Extended Release Oral Tablet [Budeprion]	40222085	Antidepressant	Depression	Drugs	
desipramine hydrochloride 150 MG Oral Tablet [Norpramin]	40237429	Antidepressant	Depression	Drugs	
escitalopram 1 MG/ML Oral Solution [Lexapro]	19102744	Antidepressant	Depression	Drugs	
desipramine hydrochloride 150 MG Oral Tablet	40237428	Antidepressant	Depression	Drugs	
{5 (milnacipran hydrochloride 12.5 MG Oral Tablet [Savella]) / 8 (milnacipran hydrochloride 25 MG Oral Tablet [Savella]) / 42 (milnacipran hydrochloride 50 MG Oral Tablet [Savella]) } Pack [Savella 4-Week Titration]	19133789	Antidepressant	Depression	Drugs	
24 HR trazodone hydrochloride 150 MG Extended Release Oral Tablet	40171493	Antidepressant	Depression	Drugs	
clomipramine hydrochloride 75 MG Oral Capsule [Anafranil]	40163077	Antidepressant	Depression	Drugs	
desipramine hydrochloride 100 MG Oral Tablet [Norpramin]	40237423	Antidepressant	Depression	Drugs	
desipramine hydrochloride 25 MG Oral Tablet [Pertofrane]	40237434	Antidepressant	Depression	Drugs	

doxepin hydrochloride 50 MG/ML Topical Cream [Zonalon]	40224911	Antidepressant	Depression	Drugs	
5-hydroxytryptophan	1363516	Antidepressant	Depression	Drugs	
clomipramine hydrochloride 50 MG Oral Capsule [Anafranil]	40163071	Antidepressant	Depression	Drugs	
amitriptyline hydrochloride 50 MG Oral Tablet [Elavil]	40162730	Antidepressant	Depression	Drugs	
vilazodone hydrochloride 40 MG Oral Tablet	40234846	Antidepressant	Depression	Drugs	
venlafaxine 50 MG Oral Tablet [Effexor]	19039820	Antidepressant	Depression	Drugs	
nortriptyline 2 MG/ML Oral Solution	19079114	Antidepressant	Depression	Drugs	
fluoxetine 4 MG/ML Oral Solution [Prozac]	19004896	Antidepressant	Depression	Drugs	
imipramine pamoate 100 MG Oral Capsule	778355	Antidepressant	Depression	Drugs	
24 HR trazodone hydrochloride 300 MG Extended Release Oral Tablet	40171495	Antidepressant	Depression	Drugs	
vortioxetine 10 MG Oral Tablet [Brintellix]	44506623	Antidepressant	Depression	Drugs	
nefazodone hydrochloride 250 MG Oral Tablet	40238429	Antidepressant	Depression	Drugs	
24 HR fluvoxamine maleate 100 MG Extended Release Oral Capsule [Luvox]	40173465	Antidepressant	Depression	Drugs	
amitriptyline hydrochloride 150 MG Oral Tablet [Endep]	40162697	Antidepressant	Depression	Drugs	
amitriptyline hydrochloride 12.5 MG / chlordiazepoxide 5 MG Oral Tablet	40162691	Antidepressant	Depression	Drugs	
Smoking Cessation 12 HR bupropion hydrochloride 150 MG Extended Release Oral Tablet [Buproban]	40221861	Antidepressant	Depression	Drugs	
alanine 11 MG/ML / arginine 8.5 MG/ML / glycine 11 MG/ML / histidine 2.6 MG/ML / isoleucine 6.2 MG/ML / leucine 8.1 MG/ML / lysine 6.24 MG/ML / methionine 3.4 MG/ML / phenylalanine 3.8 MG/ML / proline 7.5 MG/ML / serine 3.7 MG/ML / threonine 4.6 MG/ML...	19133498	Antidepressant	Depression	Drugs	
desipramine hydrochloride 25 MG Oral Tablet [Norpramin]	40237433	Antidepressant	Depression	Drugs	
milnacipran hydrochloride 50 MG Oral Tablet	19080255	Antidepressant	Depression	Drugs	
vilazodone hydrochloride 20 MG Oral Tablet	40234842	Antidepressant	Depression	Drugs	
nefazodone hydrochloride 50 MG Oral Tablet	40238435	Antidepressant	Depression	Drugs	
desipramine hydrochloride 10 MG Oral Tablet [Norpramin]	40237419	Antidepressant	Depression	Drugs	
{3 (0.2 ML) (esketamine 140 MG/ML Nasal Spray) } Pack	1366734	Antidepressant	Depression	Drugs	
paroxetine hydrochloride 2 MG/ML Oral Suspension [Paxil]	722066	Antidepressant	Depression	Drugs	
fluoxetine 90 MG Delayed Release Oral Capsule	755741	Antidepressant	Depression	Drugs	
mirtazapine 30 MG Disintegrating Oral Tablet [Remeron]	19128280	Antidepressant	Depression	Drugs	
vortioxetine 5 MG Oral Tablet [Brintellix]	44506616	Antidepressant	Depression	Drugs	
fluoxetine 50 MG / olanzapine 12 MG Oral Capsule	19102554	Antidepressant	Depression	Drugs	
milnacipran hydrochloride 100 MG Oral Tablet	19080230	Antidepressant	Depression	Drugs	
paroxetine mesylate 10 MG Oral Tablet	35604589	Antidepressant	Depression	Drugs	
bupropion hydrochloride 200 MG Extended Release Oral Tablet	40222075	Antidepressant	Depression	Drugs	
24 HR trazodone hydrochloride 300 MG Extended Release Oral Tablet [Oleptro]	40171496	Antidepressant	Depression	Drugs	
paroxetine hydrochloride 2 MG/ML Oral Suspension	722068	Antidepressant	Depression	Drugs	
mirtazapine 45 MG Disintegrating Oral Tablet [Remeron]	725208	Antidepressant	Depression	Drugs	
vortioxetine 20 MG Oral Tablet [Brintellix]	44506620	Antidepressant	Depression	Drugs	
imipramine pamoate 100 MG Oral Capsule [Tofranil-PM]	778360	Antidepressant	Depression	Drugs	
doxepin hydrochloride 10 MG/ML Oral Solution [Sinequan]	40224882	Antidepressant	Depression	Drugs	
{7 (vilazodone hydrochloride 10 MG Oral Tablet [Viibryd]) / 23 (vilazodone hydrochloride 20 MG Oral Tablet [Viibryd]) } Pack [Viibryd Starter Pack 10/20 30 Day Pack]	46234549	Antidepressant	Depression	Drugs	
desipramine hydrochloride 50 MG Oral Tablet [Norpramin]	40237442	Antidepressant	Depression	Drugs	
fluoxetine 50 MG / olanzapine 6 MG Oral Capsule	19102555	Antidepressant	Depression	Drugs	
tryptophan 500 MG Oral Capsule	19022489	Antidepressant	Depression	Drugs	
nefazodone hydrochloride 100 MG Oral Tablet [Serzone]	40238416	Antidepressant	Depression	Drugs	
sertraline 20 MG/ML Oral Solution [Zoloft]	40165283	Antidepressant	Depression	Drugs	
citalopram 2 MG/ML Oral Solution [Celexa]	797621	Antidepressant	Depression	Drugs	
{2 (0.2 ML) (esketamine 140 MG/ML Nasal Spray [Spravato]) } Pack [Spravato 56 MG Dose Kit]	1366737	Antidepressant	Depression	Drugs	
24 HR fluvoxamine maleate 100 MG Extended Release Oral Capsule	40173459	Antidepressant	Depression	Drugs	
24 HR bupropion hydrobromide 348 MG Extended Release Oral Tablet	40221879	Antidepressant	Depression	Drugs	
bupropion hydrochloride 150 MG Extended Release Oral Tablet [Zyban]	40222069	Antidepressant	Depression	Drugs	
vilazodone hydrochloride 10 MG Oral Tablet	40234838	Antidepressant	Depression	Drugs	
milnacipran hydrochloride 12.5 MG Oral Tablet [Savella]	19080254	Antidepressant	Depression	Drugs	
amoxapine 100 MG Oral Tablet	713110	Antidepressant	Depression	Drugs	
alanine 7.1 MG/ML / arginine 9.5 MG/ML / cysteine 0.16 MG/ML / glycine 14 MG/ML / histidine 2.8 MG/ML / isoleucine 6.9 MG/ML / leucine 9.1 MG/ML / lysine 7.3 MG/ML / methionine 5.3 MG/ML / phenylalanine 5.6 MG/ML / phosphoric acid 1.2 MG/ML / proline 1...	40227648	Antidepressant	Depression	Drugs	
protriptyline	754270	Antidepressant	Depression	Drugs	
paroxetine mesylate 20 MG Oral Tablet	35604702	Antidepressant	Depression	Drugs	
clomipramine hydrochloride 25 MG Oral Capsule [Anafranil]	40163064	Antidepressant	Depression	Drugs	
imipramine pamoate 150 MG Oral Capsule	778325	Antidepressant	Depression	Drugs	
fluvoxamine maleate 25 MG Oral Tablet [Luvox]	40174736	Antidepressant	Depression	Drugs	
bupropion hydrochloride 150 MG Extended Release Oral Tablet [Wellbutrin]	40222068	Antidepressant	Depression	Drugs	
venlafaxine 37.5 MG Extended Release Oral Tablet	743760	Antidepressant	Depression	Drugs	
paroxetine hydrochloride 12.5 MG Extended Release Oral Tablet	19115249	Antidepressant	Depression	Drugs	
fluoxetine 90 MG Delayed Release Oral Capsule [Prozac]	19122226	Antidepressant	Depression	Drugs	
tranylcypromine	703470	Antidepressant	Depression	Drugs	
nefazodone hydrochloride 250 MG Oral Tablet [Serzone]	40238430	Antidepressant	Depression	Drugs	
nortriptyline 10 MG Oral Capsule [Pamelor]	19041173	Antidepressant	Depression	Drugs	

alanine 10.4 MG/ML / arginine 5.75 MG/ML / glucose 150 MG/ML / glycine 5.15 MG/ML / histidine 2.4 MG/ML / isoleucine 3 MG/ML / leucine 3.65 MG/ML / lysine 2.9 MG/ML / methionine 2 MG/ML / phenylalanine 2.8 MG/ML / proline 3.4 MG/ML / serine 2.5 MG/ML /...	19130811	Antidepressant	Depression	Drugs	
amitriptyline hydrochloride 100 MG Oral Tablet [Elavil]	40162684	Antidepressant	Depression	Drugs	
fluoxetine 25 MG / olanzapine 6 MG Oral Capsule	19102552	Antidepressant	Depression	Drugs	
24 HR bupropion hydrobromide 348 MG Extended Release Oral Tablet [Aplenzin]	40221880	Antidepressant	Depression	Drugs	
amoxapine 150 MG Oral Tablet	713111	Antidepressant	Depression	Drugs	
doxepin hydrochloride 100 MG Oral Capsule [Adapin]	40224886	Antidepressant	Depression	Drugs	
alanine 8.8 MG/ML / arginine 4.89 MG/ML / glucose 250 MG/ML / glycine 4.38 MG/ML / histidine 2.04 MG/ML / isoleucine 2.55 MG/ML / leucine 3.11 MG/ML / lysine 2.47 MG/ML / methionine 1.7 MG/ML / phenylalanine 2.38 MG/ML / proline 2.89 MG/ML / serine 2.1...	19130792	Antidepressant	Depression	Drugs	
doxepin hydrochloride 50 MG/ML Topical Cream [Prudoxin]	40224910	Antidepressant	Depression	Drugs	
paroxetine hydrochloride 37.5 MG Extended Release Oral Tablet	722157	Antidepressant	Depression	Drugs	
maprotiline	794147	Antidepressant	Depression	Drugs	
maprotiline hydrochloride 50 MG Oral Tablet [Ludiomil]	42800989	Antidepressant	Depression	Drugs	
nefazodone hydrochloride 300 MG Oral Tablet	40238433	Antidepressant	Depression	Drugs	
nortriptyline 2 MG/ML Oral Solution [Aventyl]	40239275	Antidepressant	Depression	Drugs	
amitriptyline hydrochloride 10 MG/ML Injectable Solution [Elavil]	40162679	Antidepressant	Depression	Drugs	
fluoxetine 10 MG Oral Capsule [Sarafem]	755821	Antidepressant	Depression	Drugs	
fluoxetine 20 MG Oral Capsule [Sarafem]	755852	Antidepressant	Depression	Drugs	
PMDD fluoxetine 15 MG Oral Tablet	19131597	Antidepressant	Depression	Drugs	
fluoxetine 10 MG Oral Tablet [Prozac]	755734	Antidepressant	Depression	Drugs	
trimipramine 25 MG Oral Capsule [Surmontil]	19039155	Antidepressant	Depression	Drugs	
amitriptyline hydrochloride 50 MG / perphenazine 4 MG Oral Tablet [Triavil]	40162726	Antidepressant	Depression	Drugs	
isocarboxazid 10 MG Oral Tablet	19004819	Antidepressant	Depression	Drugs	
trimipramine 50 MG Oral Capsule	705797	Antidepressant	Depression	Drugs	
protriptyline hydrochloride 10 MG Oral Tablet [Vivactil]	40175377	Antidepressant	Depression	Drugs	
imipramine pamoate 75 MG Oral Capsule [Tofranil-PM]	778323	Antidepressant	Depression	Drugs	
nefazodone hydrochloride 50 MG Oral Tablet [Serzone]	40238436	Antidepressant	Depression	Drugs	
alanine 5.4 MG/ML / arginine 12 MG/ML / aspartate 3.2 MG/ML / cysteine 0.24 MG/ML / glutamate 5 MG/ML / glycine 3.6 MG/ML / histidine 4.8 MG/ML / isoleucine 8.2 MG/ML / leucine 1.4 MG/ML / lysine 1.2 MG/ML / methionine 3.4 MG/ML / phenylalanine 4.8 MG/...	40243299	Antidepressant	Depression	Drugs	
desipramine hydrochloride 75 MG Oral Tablet [Norpramin]	40237446	Antidepressant	Depression	Drugs	
imipramine pamoate 150 MG Oral Capsule [Tofranil-PM]	778326	Antidepressant	Depression	Drugs	
paroxetine hydrochloride 25 MG Extended Release Oral Tablet	722156	Antidepressant	Depression	Drugs	
alanine 11 MG/ML / arginine 8.5 MG/ML / glycine 11 MG/ML / histidine 2.6 MG/ML / isoleucine 6.2 MG/ML / leucine 8.1 MG/ML / lysine 6.24 MG/ML / magnesium chloride 0.00502 MEQ/ML / methionine 3.4 MG/ML / phenylalanine 3.8 MG/ML / potassium chloride 0.06...	40243286	Antidepressant	Depression	Drugs	
24 HR trazodone hydrochloride 150 MG Extended Release Oral Tablet [Oleptro]	40171494	Antidepressant	Depression	Drugs	
alanine 8.8 MG/ML / arginine 4.89 MG/ML / glucose 50 MG/ML / glycine 4.38 MG/ML / histidine 2.04 MG/ML / isoleucine 2.55 MG/ML / leucine 3.11 MG/ML / lysine 2.47 MG/ML / methionine 1.7 MG/ML / phenylalanine 2.38 MG/ML / proline 2.89 MG/ML / serine 2.13...	19130795	Antidepressant	Depression	Drugs	
amitriptyline hydrochloride 25 MG / chlordiazepoxide 10 MG Oral Tablet	40162701	Antidepressant	Depression	Drugs	
alanine 10.4 MG/ML / arginine 5.75 MG/ML / calcium chloride 0.004 MEQ/ML / dibasic potassium phosphate 2.61 MG/ML / glucose 200 MG/ML / glycine 5.15 MG/ML / histidine 2.4 MG/ML / isoleucine 3 MG/ML / leucine 3.65 MG/ML / lysine 2.9 MG/ML / magnesium ch...	19131089	Antidepressant	Depression	Drugs	
24 HR fluvoxamine maleate 150 MG Extended Release Oral Capsule [Luvox]	40173467	Antidepressant	Depression	Drugs	
bupropion hydrobromide 348 MG Extended Release Oral Tablet	40222100	Antidepressant	Depression	Drugs	
milnacipran hydrochloride 12.5 MG Oral Tablet	19080253	Antidepressant	Depression	Drugs	
Amitriptyline 20 MG/ML	589159	Antidepressant	Depression	Drugs	
amoxapine 25 MG Oral Tablet [Asendin]	19029977	Antidepressant	Depression	Drugs	
{7 (vilazodone hydrochloride 10 MG Oral Tablet) / 23 (vilazodone hydrochloride 20 MG Oral Tablet) } Pack	46234545	Antidepressant	Depression	Drugs	
maprotiline hydrochloride 50 MG Oral Tablet	42800988	Antidepressant	Depression	Drugs	
5-hydroxytryptophan 100 MG Oral Capsule	19103431	Antidepressant	Depression	Drugs	
fluoxetine 25 MG / olanzapine 3 MG Oral Capsule	755855	Antidepressant	Depression	Drugs	
alanine 27.6 MG/ML / arginine 19.6 MG/ML / aspartate 6 MG/ML / glutamate 10.2 MG/ML / glycine 20.6 MG/ML / histidine 11.8 MG/ML / isoleucine 10.8 MG/ML / leucine 10.8 MG/ML / lysine 13.5 MG/ML / methionine 7.6 MG/ML / phenylalanine 10 MG/ML / proline 1...	19131138	Antidepressant	Depression	Drugs	
duloxetine 30 MG [Cymbalta]	19060283	Antidepressant	Depression	Drugs	
5-hydroxytryptophan 200 MG Oral Capsule	43531880	Antidepressant	Depression	Drugs	
24 HR bupropion hydrobromide 174 MG Extended Release Oral Tablet [Aplenzin]	40221878	Antidepressant	Depression	Drugs	
bupropion hydrochloride 100 MG Extended Release Oral Tablet [Wellbutrin]	40222059	Antidepressant	Depression	Drugs	
amitriptyline hydrochloride 10 MG / perphenazine 4 MG Oral Tablet	40162665	Antidepressant	Depression	Drugs	
5-hydroxytryptophan 50 MG Oral Capsule	1363520	Antidepressant	Depression	Drugs	
amitriptyline hydrochloride 25 MG / perphenazine 2 MG Oral Tablet [Triavil]	40162707	Antidepressant	Depression	Drugs	
amitriptyline hydrochloride 10 MG / perphenazine 2 MG Oral Tablet [Triavil]	40162661	Antidepressant	Depression	Drugs	
24 HR bupropion hydrobromide 522 MG Extended Release Oral Tablet	40221881	Antidepressant	Depression	Drugs	
bupropion hydrochloride 200 MG Extended Release Oral Tablet [Wellbutrin]	40222076	Antidepressant	Depression	Drugs	
bupropion hydrochloride 300 MG Extended Release Oral Tablet [Wellbutrin]	40222086	Antidepressant	Depression	Drugs	

bupropion hydrochloride 150 MG Extended Release Oral Tablet [Buproban]	40222067	Antidepressant	Depression	Drugs	
maprotiline hydrochloride 75 MG Oral Tablet	42800992	Antidepressant	Depression	Drugs	
PMDD fluoxetine 10 MG Oral Tablet	740117	Antidepressant	Depression	Drugs	
trimipramine 100 MG Oral Capsule [Surmontil]	19039154	Antidepressant	Depression	Drugs	
amitriptyline hydrochloride 75 MG Oral Tablet [Elavil]	40162738	Antidepressant	Depression	Drugs	
nefazodone hydrochloride 200 MG Oral Tablet [Serzone]	40238424	Antidepressant	Depression	Drugs	
isocarboxazid 10 MG Oral Tablet [Marplan]	19025733	Antidepressant	Depression	Drugs	
5-hydroxytryptophan 50 MG / magnesium oxide 50 MG / melatonin 2 MG / tryptophan 100 MG / vitamin B6 10 MG Oral Capsule [Somnicin]	42799061	Antidepressant	Depression	Drugs	
maprotiline hydrochloride 25 MG Oral Tablet [Ludiomil]	42800985	Antidepressant	Depression	Drugs	
alanine 4.22 MG/ML / arginine 4.32 MG/ML / aspartate 2.98 MG/ML / glucose 200 MG/ML / glutamate 3.14 MG/ML / glycine 2.12 MG/ML / histidine 1.28 MG/ML / isoleucine 2.8 MG/ML / leucine 4.25 MG/ML / lysine 4.46 MG/ML / methionine 0.73 MG/ML / phenylalani...	19130567	Antidepressant	Depression	Drugs	
alanine 8.8 MG/ML / arginine 4.89 MG/ML / glucose 100 MG/ML / glycine 4.38 MG/ML / histidine 2.04 MG/ML / isoleucine 2.55 MG/ML / leucine 3.11 MG/ML / lysine 2.47 MG/ML / methionine 1.7 MG/ML / phenylalanine 2.38 MG/ML / proline 2.89 MG/ML / serine 2.1...	19130786	Antidepressant	Depression	Drugs	
fluoxetine 25 MG / olanzapine 6 MG Oral Capsule [Symbyax]	19127038	Antidepressant	Depression	Drugs	
duloxetine Oral Capsule	40058318	Antidepressant	Depression	Drugs	
trazodone Oral Tablet [Desyrel]	40090686	Antidepressant	Depression	Drugs	
bupropion Extended Release Oral Tablet [Wellbutrin]	40102862	Antidepressant	Depression	Drugs	
Sprinkle duloxetine 60 MG Delayed Release Oral Capsule [Drizalma]	37496769	Antidepressant	Depression	Drugs	
tryptophan 500 MG Oral Tablet	19006223	Antidepressant	Depression	Drugs	
paroxetine mesylate 40 MG Oral Tablet	35604708	Antidepressant	Depression	Drugs	
{7 (vilazodone hydrochloride 10 MG Oral Tablet) / 7 (vilazodone hydrochloride 20 MG Oral Tablet) / 16 (vilazodone hydrochloride 40 MG Oral Tablet) } Pack	40235111	Antidepressant	Depression	Drugs	
paroxetine mesylate 30 MG Oral Tablet	35604705	Antidepressant	Depression	Drugs	
24 HR bupropion hydrobromide 174 MG Extended Release Oral Tablet	40221877	Antidepressant	Depression	Drugs	
tryptophan	19006186	Antidepressant	Depression	Drugs	
tryptophan 500 MG Oral Tablet [Pacitron]	19024357	Antidepressant	Depression	Drugs	
amoxapine 100 MG Oral Tablet [Asendin]	19029947	Antidepressant	Depression	Drugs	
amitriptyline hydrochloride 12.5 MG / chlordiazepoxide 5 MG Oral Tablet [Limbitrol]	40162692	Antidepressant	Depression	Drugs	
amoxapine 50 MG Oral Tablet [Asendin]	19029980	Antidepressant	Depression	Drugs	
fluoxetine 50 MG / olanzapine 6 MG Oral Capsule [Symbyax]	19127042	Antidepressant	Depression	Drugs	
Sprinkle duloxetine 20 MG Delayed Release Oral Capsule [Drizalma]	37496760	Antidepressant	Depression	Drugs	
trimipramine 25 MG Oral Capsule	705796	Antidepressant	Depression	Drugs	
amoxapine 150 MG Oral Tablet [Asendin]	19029974	Antidepressant	Depression	Drugs	
amitriptyline hydrochloride 25 MG / perphenazine 4 MG Oral Tablet	40162711	Antidepressant	Depression	Drugs	
maprotiline hydrochloride 75 MG Oral Tablet [Ludiomil]	42800993	Antidepressant	Depression	Drugs	
alanine 10.4 MG/ML / arginine 5.75 MG/ML / calcium chloride 0.004 MEQ/ML / dibasic potassium phosphate 2.61 MG/ML / glucose 350 MG/ML / glycine 5.15 MG/ML / histidine 2.4 MG/ML / isoleucine 3 MG/ML / leucine 3.65 MG/ML / magnesium chloride 0.01 MEQ/ML...	19131095	Antidepressant	Depression	Drugs	
alanine 2.1 MG/ML / arginine 2.9 MG/ML / calcium acetate 0.003 MEQ/ML / cysteine 0.2 MG/ML / glycerin 30 MG/ML / glycine 4.2 MG/ML / histidine 0.85 MG/ML / isoleucine 2.1 MG/ML / leucine 2.7 MG/ML / lysine 3.1 MG/ML / magnesium acetate 0.008 MEQ/ML / m...	19131215	Antidepressant	Depression	Drugs	
alanine 8.8 MG/ML / arginine 4.89 MG/ML / calcium chloride 0.004 MEQ/ML / dibasic potassium phosphate 2.61 MG/ML / glucose 50 MG/ML / glycine 4.38 MG/ML / histidine 2.04 MG/ML / isoleucine 2.55 MG/ML / leucine 3.11 MG/ML / lysine 2.47 MG/ML / magnesium...	19131062	Antidepressant	Depression	Drugs	
alanine 8.8 MG/ML / arginine 4.89 MG/ML / glucose 50 MG/ML / glycine 4.38 MG/ML / histidine 2.04 MG/ML / isoleucine 2.55 MG/ML / leucine 3.11 MG/ML / lysine 2.47 MG/ML / methionine 1.7 MG/ML / phenylalanine 2.38 MG/ML / proline 2.89 MG/ML / serine 2.13...	19130793	Antidepressant	Depression	Drugs	
imipramine hydrochloride 25 MG Oral Tablet [Pramimil]	19000966	Antidepressant	Depression	Drugs	
citalopram 10 MG Oral Tablet [Cipramil]	19017328	Antidepressant	Depression	Drugs	
imipramine pamoate 125 MG Oral Capsule	778353	Antidepressant	Depression	Drugs	
bupropion Extended Release Oral Tablet [Buproban]	40125347	Antidepressant	Depression	Drugs	
alanine 2.1 MG/ML / arginine 2.9 MG/ML / calcium acetate 0.003 MEQ/ML / cysteine 0.2 MG/ML / glycerin 30 MG/ML / glycine 4.2 MG/ML / histidine 0.85 MG/ML / isoleucine 2.1 MG/ML / leucine 2.7 MG/ML / lysine 3.1 MG/ML / magnesium acetate 0.008 MEQ/ML / m...	19131217	Antidepressant	Depression	Drugs	
alanine 21.7 MG/ML / arginine 14.7 MG/ML / aspartate 4.34 MG/ML / glutamate 7.49 MG/ML / glycine 10.4 MG/ML / histidine 8.94 MG/ML / isoleucine 7.49 MG/ML / leucine 10.4 MG/ML / lysine 11.8 MG/ML / methionine 7.49 MG/ML / phenylalanine 10.4 MG/ML / pro...	45892124	Antidepressant	Depression	Drugs	
citalopram 20 MG Oral Tablet [Cipramil]	19010672	Antidepressant	Depression	Drugs	
alanine 10.4 MG/ML / arginine 5.75 MG/ML / calcium chloride 0.004 MEQ/ML / dibasic potassium phosphate 2.61 MG/ML / glucose 150 MG/ML / glycine 5.15 MG/ML / histidine 2.4 MG/ML / isoleucine 3 MG/ML / leucine 3.65 MG/ML / lysine 2.9 MG/ML / magnesium ch...	19131080	Antidepressant	Depression	Drugs	
alanine 20.7 MG/ML / arginine 11.5 MG/ML / glycine 10.3 MG/ML / histidine 4.8 MG/ML / isoleucine 6 MG/ML / leucine 7.3 MG/ML / lysine 5.8 MG/ML / methionine 4 MG/ML / phenylalanine 5.6 MG/ML / proline 6.8 MG/ML / serine 5 MG/ML / threonine 4.2 MG/ML /...	19131341	Antidepressant	Depression	Drugs	

alanine 12.8 MG/ML / arginine 9.8 MG/ML / glycine 12.8 MG/ML / histidine 3 MG/ML / isoleucine 7.2 MG/ML / leucine 9.4 MG/ML / lysine 7.2 MG/ML / methionine 4 MG/ML / phenylalanine 4.4 MG/ML / proline 8.6 MG/ML / serine 4.2 MG/ML / threonine 5.2 MG/ML /...	19133481	Antidepressant	Depression	Drugs	
imipramine hydrochloride 10 MG Oral Tablet [Pramimil]	19000965	Antidepressant	Depression	Drugs	
alanine 10.4 MG/ML / arginine 5.75 MG/ML / calcium chloride 0.004 MEQ/ML / dibasic potassium phosphate 2.61 MG/ML / glucose 200 MG/ML / glycine 5.15 MG/ML / histidine 2.4 MG/ML / isoleucine 3 MG/ML / leucine 3.65 MG/ML / lysine 2.9 MG/ML / magnesium ch...	19131087	Antidepressant	Depression	Drugs	
alanine 5.7 MG/ML / arginine 3.16 MG/ML / calcium chloride 0.004 MEQ/ML / dibasic potassium phosphate 2.61 MG/ML / glucose 50 MG/ML / glycine 2.83 MG/ML / histidine 1.32 MG/ML / isoleucine 1.65 MG/ML / leucine 2.01 MG/ML / lysine 1.59 MG/ML / magnesium...	19131071	Antidepressant	Depression	Drugs	
duloxetine 20 MG Oral Capsule	19112657	Antidepressant	Depression	Drugs	
vortioxetine Oral Tablet [Trintellix]	42629568	Antidepressant	Depression	Drugs	
alanine 8.44 MG/ML / arginine 8.65 MG/ML / aspartate 5.95 MG/ML / glutamate 6.27 MG/ML / glycine 4.25 MG/ML / histidine 2.55 MG/ML / isoleucine 5.61 MG/ML / leucine 8.5 MG/ML / lysine 8.93 MG/ML / methionine 1.46 MG/ML / phenylalanine 2.53 MG/ML / prol...	19130651	Antidepressant	Depression	Drugs	
venlafaxine 75 MG [Effexor]	19118743	Antidepressant	Depression	Drugs	
amitriptyline 30 MG	19109687	Antidepressant	Depression	Drugs	
clomipramine hydrochloride 50 MG	40163069	Antidepressant	Depression	Drugs	
doxepin 100 MG	19085200	Antidepressant	Depression	Drugs	
nortriptyline 50 MG [Pamelor]	19026384	Antidepressant	Depression	Drugs	
mirtazapine 45 MG [Remeron]	19015749	Antidepressant	Depression	Drugs	
trazodone hydrochloride 50 MG	40163471	Antidepressant	Depression	Drugs	
trazodone hydrochloride 100 MG	40163458	Antidepressant	Depression	Drugs	
fluoxetine 40 MG [Prozac]	19080284	Antidepressant	Depression	Drugs	
fluoxetine 20 MG [Prozac]	19080281	Antidepressant	Depression	Drugs	
doxepin 10 MG	738240	Antidepressant	Depression	Drugs	
fluoxetine 60 MG	19091121	Antidepressant	Depression	Drugs	
amitriptyline hydrochloride 50 MG	40162724	Antidepressant	Depression	Drugs	
escitalopram 20 MG [Lexapro]	19033450	Antidepressant	Depression	Drugs	
sertraline 50 MG [Zoloft]	19043842	Antidepressant	Depression	Drugs	
bupropion hydrochloride 100 MG [Wellbutrin]	40222063	Antidepressant	Depression	Drugs	
sertraline 100 MG	19083100	Antidepressant	Depression	Drugs	
bupropion hydrochloride 200 MG [Wellbutrin]	40222077	Antidepressant	Depression	Drugs	
nefazodone hydrochloride 150 MG	40238418	Antidepressant	Depression	Drugs	
clomipramine hydrochloride 25 MG [Anafranil]	40163066	Antidepressant	Depression	Drugs	
nortriptyline 25 MG	19083195	Antidepressant	Depression	Drugs	
imipramine hydrochloride 25 MG	778295	Antidepressant	Depression	Drugs	
nortriptyline 10 MG	721761	Antidepressant	Depression	Drugs	
clomipramine hydrochloride 50 MG [Anafranil]	40163073	Antidepressant	Depression	Drugs	
bupropion hydrochloride 150 MG	40222064	Antidepressant	Depression	Drugs	
doxepin 50 MG	738262	Antidepressant	Depression	Drugs	
fluvoxamine maleate 100 MG [Luvox]	40174727	Antidepressant	Depression	Drugs	
mirtazapine 30 MG [Remeron]	19015748	Antidepressant	Depression	Drugs	
venlafaxine 37.5 MG [Effexor]	19021629	Antidepressant	Depression	Drugs	
escitalopram 10 MG [Lexapro]	19033449	Antidepressant	Depression	Drugs	
bupropion hydrochloride 300 MG	40222083	Antidepressant	Depression	Drugs	
bupropion hydrochloride 150 MG [Wellbutrin]	40222072	Antidepressant	Depression	Drugs	
citalopram 20 MG [Celexa]	19120296	Antidepressant	Depression	Drugs	
nortriptyline 30 MG	721796	Antidepressant	Depression	Drugs	
sertraline 25 MG	739282	Antidepressant	Depression	Drugs	
bupropion hydrochloride 75 MG [Wellbutrin]	40222094	Antidepressant	Depression	Drugs	
doxepin 25 MG	738241	Antidepressant	Depression	Drugs	
duloxetine 20 MG [Cymbalta]	19060282	Antidepressant	Depression	Drugs	
venlafaxine 150 MG [Effexor]	19127359	Antidepressant	Depression	Drugs	
amitriptyline 25 MG	19085723	Antidepressant	Depression	Drugs	
citalopram 10 MG [Celexa]	19120749	Antidepressant	Depression	Drugs	
sertraline 100 MG [Zoloft]	19043841	Antidepressant	Depression	Drugs	
sertraline 25 MG [Zoloft]	19043843	Antidepressant	Depression	Drugs	
imipramine hydrochloride 50 MG	778298	Antidepressant	Depression	Drugs	
nortriptyline 75 MG	721794	Antidepressant	Depression	Drugs	
imipramine hydrochloride 10 MG	778356	Antidepressant	Depression	Drugs	
mirtazapine 45 MG	725203	Antidepressant	Depression	Drugs	
fluoxetine 10 MG	755762	Antidepressant	Depression	Drugs	
amitriptyline 75 MG	710183	Antidepressant	Depression	Drugs	
amitriptyline 10 MG	710185	Antidepressant	Depression	Drugs	
amitriptyline hydrochloride 25 MG [Elavil]	40162720	Antidepressant	Depression	Drugs	
nefazodone hydrochloride 200 MG [Serzone]	40238425	Antidepressant	Depression	Drugs	
duloxetine 60 MG [Cymbalta]	19060284	Antidepressant	Depression	Drugs	
citalopram 40 MG	797637	Antidepressant	Depression	Drugs	
bupropion hydrochloride 150 MG [Zyban]	40222073	Antidepressant	Depression	Drugs	

fluoxetine 40 MG	19084977	Antidepressant	Depression	Drugs	
bupropion hydrochloride 300 MG [Wellbutrin]	40222088	Antidepressant	Depression	Drugs	
citalopram 40 MG [Celexa]	19058160	Antidepressant	Depression	Drugs	
trazodone hydrochloride 150 MG [Desyrel]	40163466	Antidepressant	Depression	Drugs	
bupropion hydrochloride 100 MG	40222056	Antidepressant	Depression	Drugs	
amitriptyline hydrochloride 100 MG	40162682	Antidepressant	Depression	Drugs	
bupropion hydrochloride 200 MG	40222074	Antidepressant	Depression	Drugs	
fluoxetine 20 MG	755763	Antidepressant	Depression	Drugs	
mirtazapine 15 MG [Remeron]	19015747	Antidepressant	Depression	Drugs	
mirtazapine 15 MG	19095236	Antidepressant	Depression	Drugs	
clomipramine hydrochloride 25 MG	40163062	Antidepressant	Depression	Drugs	
nefazodone hydrochloride 150 MG [Serzone]	40238421	Antidepressant	Depression	Drugs	
trimipramine	705755	Antidepressant	Depression	Drugs	
fluoxetine 15 MG	19131596	Antidepressant	Depression	Drugs	
imipramine hydrochloride 150 MG	19135757	Antidepressant	Depression	Drugs	
sertraline 200 MG	739283	Antidepressant	Depression	Drugs	
nortriptyline 25 MG [Pamelor]	19118843	Antidepressant	Depression	Drugs	
nortriptyline 75 MG [Pamelor]	19118855	Antidepressant	Depression	Drugs	
protriptyline hydrochloride 10 MG [Vivactil]	40175378	Antidepressant	Depression	Drugs	
desipramine 50 MG	19082930	Antidepressant	Depression	Drugs	
bupropion hydrochloride 50 MG	40222089	Antidepressant	Depression	Drugs	
fluvoxamine maleate 50 MG [Luvox]	40174741	Antidepressant	Depression	Drugs	
protriptyline hydrochloride 10 MG	40175375	Antidepressant	Depression	Drugs	
fluoxetine 20 MG [Sarafem]	19090639	Antidepressant	Depression	Drugs	
imipramine hydrochloride 10 MG [Tofranil]	19001275	Antidepressant	Depression	Drugs	
nortriptyline 50 MG	721793	Antidepressant	Depression	Drugs	
bupropion hydrochloride 100 MG [Budeprion]	40222062	Antidepressant	Depression	Drugs	
amitriptyline hydrochloride 10 MG [Elavil]	40162675	Antidepressant	Depression	Drugs	
nefazodone hydrochloride 100 MG [Serzone]	40238417	Antidepressant	Depression	Drugs	
imipramine hydrochloride 75 MG	19135759	Antidepressant	Depression	Drugs	
duloxetine 30 MG	715296	Antidepressant	Depression	Drugs	
amitriptyline hydrochloride 150 MG	40162694	Antidepressant	Depression	Drugs	
fluvoxamine maleate 150 MG	40174728	Antidepressant	Depression	Drugs	
clomipramine hydrochloride 20 MG	40169581	Antidepressant	Depression	Drugs	
fluoxetine 50 MG	19103071	Antidepressant	Depression	Drugs	
desipramine 25 MG	19082929	Antidepressant	Depression	Drugs	
trazodone hydrochloride 50 MG [Desyrel]	40163475	Antidepressant	Depression	Drugs	
trazodone 75 MG	703615	Antidepressant	Depression	Drugs	
trazodone hydrochloride 150 MG	40163463	Antidepressant	Depression	Drugs	
fluvoxamine 50 MG	751419	Antidepressant	Depression	Drugs	
trazodone 25 MG	19109758	Antidepressant	Depression	Drugs	
fluoxetine 10 MG [Prozac]	19080283	Antidepressant	Depression	Drugs	
fluoxetine 60 MG [Prozac]	19116858	Antidepressant	Depression	Drugs	
bupropion hydrochloride 150 MG [Budeprion]	40222070	Antidepressant	Depression	Drugs	
escitalopram 5 MG [Lexapro]	19121118	Antidepressant	Depression	Drugs	
mirtazapine 30 MG	19095237	Antidepressant	Depression	Drugs	
nortriptyline 20 MG	721797	Antidepressant	Depression	Drugs	
citalopram 20 MG	797636	Antidepressant	Depression	Drugs	
doxepin 75 MG	738263	Antidepressant	Depression	Drugs	
citalopram 10 MG	797639	Antidepressant	Depression	Drugs	
fluvoxamine maleate 150 MG [Luvox]	40174733	Antidepressant	Depression	Drugs	
sertraline 50 MG	739211	Antidepressant	Depression	Drugs	
venlafaxine 150 MG	743759	Antidepressant	Depression	Drugs	
fluvoxamine maleate 25 MG	40174734	Antidepressant	Depression	Drugs	
sertraline 150 MG	739284	Antidepressant	Depression	Drugs	
fluoxetine 4 MG/ML [Prozac]	19080282	Antidepressant	Depression	Drugs	
nefazodone hydrochloride 250 MG [Serzone]	40238431	Antidepressant	Depression	Drugs	
venlafaxine 225 MG	19131402	Antidepressant	Depression	Drugs	
clomipramine hydrochloride 75 MG [Anafranil]	40163078	Antidepressant	Depression	Drugs	
amitriptyline hydrochloride 50 MG [Elavil]	40162733	Antidepressant	Depression	Drugs	
sertraline 20 MG/ML [Zoloft]	40165284	Antidepressant	Depression	Drugs	
venlafaxine 37.5 MG	743755	Antidepressant	Depression	Drugs	
tranylcypromine 10 MG [Parnate]	19115965	Antidepressant	Depression	Drugs	
fluvoxamine 100 MG	751420	Antidepressant	Depression	Drugs	
imipramine hydrochloride 25 MG [Tofranil]	19001272	Antidepressant	Depression	Drugs	
venlafaxine 75 MG	19083160	Antidepressant	Depression	Drugs	
nefazodone hydrochloride 300 MG	40238432	Antidepressant	Depression	Drugs	
protriptyline hydrochloride 5 MG	40175379	Antidepressant	Depression	Drugs	
desipramine hydrochloride 100 MG	40237421	Antidepressant	Depression	Drugs	
nortriptyline 10 MG [Pamelor]	19026382	Antidepressant	Depression	Drugs	
desipramine 40 MG	717063	Antidepressant	Depression	Drugs	
trazodone hydrochloride 300 MG [Desyrel]	40163470	Antidepressant	Depression	Drugs	

desipramine dibudinate 30 MG	40237415	Antidepressant	Depression	Drugs	
desipramine hydrochloride 10 MG	40237417	Antidepressant	Depression	Drugs	
fluoxetine 90 MG	755766	Antidepressant	Depression	Drugs	
bupropion hydrochloride 450 MG	42705084	Antidepressant	Depression	Drugs	
sertraline Oral Tablet	40081943	Antidepressant	Depression	Drugs	
duloxetine 20 MG	715293	Antidepressant	Depression	Drugs	
nefazodone hydrochloride 50 MG [Serzone]	40238437	Antidepressant	Depression	Drugs	
trazodone hydrochloride 300 MG	40163467	Antidepressant	Depression	Drugs	
fluoxetine 10 MG [Sarafem]	19090638	Antidepressant	Depression	Drugs	
trazodone 10 MG/ML	703614	Antidepressant	Depression	Drugs	
nortriptyline 40 MG	721798	Antidepressant	Depression	Drugs	
isocarboxazid	781705	Antidepressant	Depression	Drugs	
doxepin 40 MG/ML	19109670	Antidepressant	Depression	Drugs	
venlafaxine 25 MG [Effexor]	19021628	Antidepressant	Depression	Drugs	
venlafaxine 50 MG [Effexor]	19118741	Antidepressant	Depression	Drugs	
fluoxetine 90 MG [Prozac]	19113117	Antidepressant	Depression	Drugs	
amitriptyline hydrochloride 5 MG/ML	40162722	Antidepressant	Depression	Drugs	
fluvoxamine maleate 100 MG	40174722	Antidepressant	Depression	Drugs	
imipramine pamoate 100 MG	19134044	Antidepressant	Depression	Drugs	
venlafaxine 100 MG [Effexor]	19021627	Antidepressant	Depression	Drugs	
amitriptyline hydrochloride 12.5 MG	40162688	Antidepressant	Depression	Drugs	
protriptyline hydrochloride 5 MG [Vivactil]	40175382	Antidepressant	Depression	Drugs	
fluoxetine 25 MG / olanzapine 12 MG Oral Capsule	19102553	Antidepressant	Depression	Drugs	
nefazodone hydrochloride 200 MG	40238422	Antidepressant	Depression	Drugs	
citalopram 2 MG/ML [Celexa]	19058161	Antidepressant	Depression	Drugs	
imipramine pamoate 125 MG	778352	Antidepressant	Depression	Drugs	
phenelzine 15 MG [Nardil]	19048255	Antidepressant	Depression	Drugs	
clomipramine 75 MG	19085292	Antidepressant	Depression	Drugs	
doxepin hydrochloride 150 MG	40224890	Antidepressant	Depression	Drugs	
desipramine hydrochloride 150 MG	40237427	Antidepressant	Depression	Drugs	
amitriptyline 15 MG	710263	Antidepressant	Depression	Drugs	
fluvoxamine maleate 150 MG Extended Release Oral Capsule	40174729	Antidepressant	Depression	Drugs	
trazodone hydrochloride 100 MG [Desyrel]	40163462	Antidepressant	Depression	Drugs	
fluoxetine 50 MG / olanzapine 12 MG Oral Capsule [Symbyax]	19127040	Antidepressant	Depression	Drugs	
trazodone Oral Solution	40090682	Antidepressant	Depression	Drugs	
Sprinkle duloxetine 30 MG Delayed Release Oral Capsule [Drizalma]	37496763	Antidepressant	Depression	Drugs	
alanine 9.93 MG/ML / arginine 10.2 MG/ML / aspartate 7 MG/ML / glutamate 7.38 MG/ML / glycine 5 MG/ML / histidine 3 MG/ML / isoleucine 6.6 MG/ML / leucine 10 MG/ML / lysine 10.5 MG/ML / methionine 1.72 MG/ML / phenylalanine 2.98 MG/ML / proline 7.22 MG...	19130670	Antidepressant	Depression	Drugs	
maprotiline hydrochloride 25 MG	42800983	Antidepressant	Depression	Drugs	
bupropion hydrochloride 75 MG	40222091	Antidepressant	Depression	Drugs	
escitalopram 10 MG	19098080	Antidepressant	Depression	Drugs	
esketamine 140 MG/ML Nasal Spray	1366618	Antidepressant	Depression	Drugs	
escitalopram 20 MG	715968	Antidepressant	Depression	Drugs	
mirtazapine 7.5 MG	19112587	Antidepressant	Depression	Drugs	
duloxetine 60 MG	715299	Antidepressant	Depression	Drugs	
desipramine hydrochloride 75 MG	40237444	Antidepressant	Depression	Drugs	
amitriptyline 75 MG [Elavil]	19117641	Antidepressant	Depression	Drugs	
paroxetine Oral Tablet	40071301	Antidepressant	Depression	Drugs	
doxepin hydrochloride 25 MG [Sinequan]	40224901	Antidepressant	Depression	Drugs	
amitriptyline hydrochloride 10 MG Oral Tablet [Endep]	40162674	Antidepressant	Depression	Drugs	
Sprinkle duloxetine 40 MG Delayed Release Oral Capsule [Drizalma]	37496766	Antidepressant	Depression	Drugs	
bupropion hydrochloride 300 MG [Budeprion]	40222087	Antidepressant	Depression	Drugs	
amitriptyline 60 MG	19110100	Antidepressant	Depression	Drugs	
fluvoxamine maleate 25 MG [Luvox]	40174737	Antidepressant	Depression	Drugs	
venlafaxine 100 MG	743754	Antidepressant	Depression	Drugs	
citalopram 2 MG/ML	19084879	Antidepressant	Depression	Drugs	
venlafaxine 25 MG	19086246	Antidepressant	Depression	Drugs	
maprotiline hydrochloride 50 MG [Ludiomil]	42800990	Antidepressant	Depression	Drugs	
amitriptyline 90 MG	19109688	Antidepressant	Depression	Drugs	
maprotiline hydrochloride 75 MG	42800991	Antidepressant	Depression	Drugs	
nortriptyline 10 MG Oral Tablet	721728	Antidepressant	Depression	Drugs	
alanine 9.93 MG/ML / arginine 10.2 MG/ML / aspartate 7 MG/ML / glutamate 7.38 MG/ML / glycine 5 MG/ML / histidine 3 MG/ML / isoleucine 6.6 MG/ML / leucine 10 MG/ML / lysine 10.5 MG/ML / methionine 1.72 MG/ML / phenylalanine 2.98 MG/ML / proline 7.22 MG...	19130668	Antidepressant	Depression	Drugs	
tryptophan Oral Tablet	40088846	Antidepressant	Depression	Drugs	
nefazodone hydrochloride 100 MG	40238414	Antidepressant	Depression	Drugs	
amitriptyline 2 MG/ML	19087006	Antidepressant	Depression	Drugs	
alanine 21.7 MG/ML / arginine 14.7 MG/ML / aspartate 4.34 MG/ML / glutamate 7.49 MG/ML / glycine 10.4 MG/ML / histidine 8.94 MG/ML / isoleucine 7.49 MG/ML / leucine 10.4 MG/ML / lysine 11.8 MG/ML / methionine 7.49 MG/ML / phenylalanine 10.4 MG/ML / pro...	19130881	Antidepressant	Depression	Drugs	

nortriptyline 25 MG [Aventyl]	19115958	Antidepressant	Depression	Drugs	
fluoxetine 6 MG	755770	Antidepressant	Depression	Drugs	
nefazodone hydrochloride 25 MG	40238426	Antidepressant	Depression	Drugs	
fluoxetine 25 MG	19103068	Antidepressant	Depression	Drugs	
fluoxetine hydrochloride 16 MG	40174701	Antidepressant	Depression	Drugs	
fluoxetine 25 MG / olanzapine 3 MG Oral Capsule [Symbyax]	19127046	Antidepressant	Depression	Drugs	
desvenlafaxine 100 MG Extended Release Oral Tablet [Pristiq]	19134912	Antidepressant	Depression	Drugs	
alanine 5.4 MG/ML / arginine 12 MG/ML / aspartate 3.2 MG/ML / cysteine 0.24 MG/ML / glutamate 5 MG/ML / glycine 3.6 MG/ML / histidine 4.8 MG/ML / isoleucine 8.2 MG/ML / leucine 1.4 MG/ML / lysine 1.2 MG/ML / methionine 3.4 MG/ML / phenylalanine 4.8 MG/...	19130902	Antidepressant	Depression	Drugs	
doxepin 5 MG	19109695	Antidepressant	Depression	Drugs	
clomipramine hydrochloride 10 MG	40163057	Antidepressant	Depression	Drugs	
nefazodone hydrochloride 50 MG	40238434	Antidepressant	Depression	Drugs	
venlafaxine 50 MG	19083159	Antidepressant	Depression	Drugs	
amitriptyline 120 MG	19109686	Antidepressant	Depression	Drugs	
bupropion	750982	Antidepressant	Depression	Drugs	
trazodone	703547	Antidepressant	Depression	Drugs	
citalopram	797617	Antidepressant	Depression	Drugs	
duloxetine	715259	Antidepressant	Depression	Drugs	
fluoxetine	755695	Antidepressant	Depression	Drugs	
paroxetine	722031	Antidepressant	Depression	Drugs	
sertraline	739138	Antidepressant	Depression	Drugs	
mirtazapine	725131	Antidepressant	Depression	Drugs	
venlafaxine	743670	Antidepressant	Depression	Drugs	
escitalopram	715939	Antidepressant	Depression	Drugs	
amitriptyline	710062	Antidepressant	Depression	Drugs	
nortriptyline	721724	Antidepressant	Depression	Drugs	
citalopram 10 MG Oral Tablet	797632	Antidepressant	Depression	Drugs	
citalopram 20 MG Oral Tablet	19023636	Antidepressant	Depression	Drugs	
citalopram 40 MG Oral Tablet	19075394	Antidepressant	Depression	Drugs	
sertraline 25 MG Oral Tablet	19079497	Antidepressant	Depression	Drugs	
sertraline 50 MG Oral Tablet	739209	Antidepressant	Depression	Drugs	
fluoxetine 10 MG Oral Capsule	19077462	Antidepressant	Depression	Drugs	
fluoxetine 20 MG Oral Capsule	19077463	Antidepressant	Depression	Drugs	
fluoxetine 40 MG Oral Capsule	755739	Antidepressant	Depression	Drugs	
mirtazapine 15 MG Oral Tablet	725178	Antidepressant	Depression	Drugs	
mirtazapine 30 MG Oral Tablet	725180	Antidepressant	Depression	Drugs	
sertraline 100 MG Oral Tablet	739207	Antidepressant	Depression	Drugs	
escitalopram 10 MG Oral Tablet	715940	Antidepressant	Depression	Drugs	
escitalopram 20 MG Oral Tablet	19098166	Antidepressant	Depression	Drugs	
nortriptyline 10 MG Oral Capsule	19019412	Antidepressant	Depression	Drugs	
nortriptyline 25 MG Oral Capsule	721760	Antidepressant	Depression	Drugs	
sertraline 50 MG Oral Tablet [Zoloft]	19037684	Antidepressant	Depression	Drugs	
sertraline 100 MG Oral Tablet [Zoloft]	19037642	Antidepressant	Depression	Drugs	
trazodone hydrochloride 50 MG Oral Tablet	40163473	Antidepressant	Depression	Drugs	
paroxetine hydrochloride 20 MG Oral Tablet	35604576	Antidepressant	Depression	Drugs	
paroxetine hydrochloride 40 MG Oral Tablet	35604586	Antidepressant	Depression	Drugs	
trazodone hydrochloride 100 MG Oral Tablet	40163460	Antidepressant	Depression	Drugs	
trazodone hydrochloride 150 MG Oral Tablet	40163464	Antidepressant	Depression	Drugs	
amitriptyline hydrochloride 10 MG Oral Tablet	40162672	Antidepressant	Depression	Drugs	
amitriptyline hydrochloride 25 MG Oral Tablet	40162717	Antidepressant	Depression	Drugs	
amitriptyline hydrochloride 50 MG Oral Tablet	40162729	Antidepressant	Depression	Drugs	
duloxetine 20 MG Delayed Release Oral Capsule	715294	Antidepressant	Depression	Drugs	
duloxetine 30 MG Delayed Release Oral Capsule	19122148	Antidepressant	Depression	Drugs	
duloxetine 60 MG Delayed Release Oral Capsule	715300	Antidepressant	Depression	Drugs	
24 HR venlafaxine 75 MG Extended Release Oral Capsule	743721	Antidepressant	Depression	Drugs	
24 HR venlafaxine 150 MG Extended Release Oral Capsule	743717	Antidepressant	Depression	Drugs	
24 HR venlafaxine 37.5 MG Extended Release Oral Capsule	743719	Antidepressant	Depression	Drugs	
duloxetine 60 MG Delayed Release Oral Capsule [Cymbalta]	715301	Antidepressant	Depression	Drugs	
12 HR bupropion hydrochloride 100 MG Extended Release Oral Tablet	40221856	Antidepressant	Depression	Drugs	
12 HR bupropion hydrochloride 150 MG Extended Release Oral Tablet	40221859	Antidepressant	Depression	Drugs	
24 HR bupropion hydrochloride 150 MG Extended Release Oral Tablet	40221871	Antidepressant	Depression	Drugs	
24 HR bupropion hydrochloride 300 MG Extended Release Oral Tablet	40221874	Antidepressant	Depression	Drugs	
Recurrent major depressive episodes, moderate	432883	Depression	Depression	Conditions	Excluded
Recurrent major depressive episodes	432285	Depression	Depression	Conditions	Excluded
Recurrent major depression in partial remission	4141454	Depression	Depression	Conditions	Excluded
Depressive disorder	440383	Depression	Depression	Conditions	
Recurrent major depression in remission	433991	Depression	Depression	Conditions	Excluded
Bipolar affective disorder, current episode depression	439254	Depression	Depression	Conditions	Excluded
Major depression, single episode	4282096	Depression	Depression	Conditions	Excluded
Bipolar affective disorder, currently depressed, in full remission	439251	Depression	Depression	Conditions	Excluded

Bipolar affective disorder, currently depressed, moderate	437528	Depression	Depression	Conditions	
Schizoaffective disorder, depressive type	4224940	Depression	Depression	Conditions	Excluded
Dysthymia	433440	Depression	Depression	Conditions	
Recurrent major depression in full remission	4263748	Depression	Depression	Conditions	Excluded
Single episode of major depression in full remission	4025677	Depression	Depression	Conditions	Excluded
Recurrent major depressive episodes, severe, with psychosis	434911	Depression	Depression	Conditions	Excluded
Chronic depression	4103574	Depression	Depression	Conditions	
Severe depression	4149321	Depression	Depression	Conditions	
Premenstrual dysphoric disorder	4242733	Depression	Depression	Conditions	Excluded
Recurrent depression	4098302	Depression	Depression	Conditions	
Moderate major depression, single episode	4049623	Depression	Depression	Conditions	Excluded
Mild major depression, single episode	4195572	Depression	Depression	Conditions	Excluded
Mild recurrent major depression	4228802	Depression	Depression	Conditions	
Recurrent major depression	4282316	Depression	Depression	Conditions	
Positive screening for depression on PHQ-9 (Patient Health Questionnaire 9)	43021839	Depression	Depression	Conditions	
Recurrent major depressive episodes, mild	438998	Depression	Depression	Conditions	Excluded
Severe major depression, single episode, with psychotic features	438406	Depression	Depression	Conditions	Excluded
Severe major depression, single episode, without psychotic features	441534	Depression	Depression	Conditions	Excluded
Moderate recurrent major depression	4077577	Depression	Depression	Conditions	
Bipolar affective disorder, currently depressed, mild	439253	Depression	Depression	Conditions	
Psychosis and severe depression co-occurrent and due to bipolar affective disorder	35622934	Depression	Depression	Conditions	
Severe recurrent major depression without psychotic features	435220	Depression	Depression	Conditions	
Mixed anxiety and depressive disorder	4338031	Depression	Depression	Conditions	
Reactive depression (situational)	4314692	Depression	Depression	Conditions	Excluded
Major depression single episode, in partial remission	4323418	Depression	Depression	Conditions	Excluded
Major depressive disorder	4152280	Depression	Depression	Conditions	
Severe recurrent major depression with psychotic features	4154309	Depression	Depression	Conditions	
Major depression in partial remission	4148630	Depression	Depression	Conditions	Excluded
Major depression in remission	4176002	Depression	Depression	Conditions	Excluded
Severe recurrent major depression	43531624	Depression	Depression	Conditions	
Single major depressive episode, severe, with psychosis	439259	Depression	Depression	Conditions	Excluded
Atypical depressive disorder	438727	Depression	Depression	Conditions	Excluded
Major depression with psychotic features	37111697	Depression	Depression	Conditions	
Mild major depression	4336957	Depression	Depression	Conditions	
Reactive depressive psychosis	435520	Depression	Depression	Conditions	Excluded
Major depression in full remission	4269493	Depression	Depression	Conditions	Excluded
Depressive disorder in remission	44782943	Depression	Depression	Conditions	Excluded
Moderate major depression	4307111	Depression	Depression	Conditions	
Menopausal depression	4223090	Depression	Depression	Conditions	
Severe major depression, single episode	42872411	Depression	Depression	Conditions	Excluded
Seasonal affective disorder	4092239	Depression	Depression	Conditions	Excluded
Recurrent major depressive disorder with postpartum onset	4324959	Depression	Depression	Conditions	
Severe major depression with psychotic features, mood-congruent	4144233	Depression	Depression	Conditions	
Multi-infarct dementia with depression	443864	Depression	Depression	Conditions	
Chronic depressive personality disorder	40481798	Depression	Depression	Conditions	
Moderately severe recurrent major depression	36714998	Depression	Depression	Conditions	
Depression screening positive	762504	Depression	Depression	Conditions	
Severe major depression with psychotic features	4250023	Depression	Depression	Conditions	
Endogenous depression	4114950	Depression	Depression	Conditions	
Mild depression	4149320	Depression	Depression	Conditions	
Acute depression	37016718	Depression	Depression	Conditions	
Severe postnatal depression	4129184	Depression	Depression	Conditions	
Moderately severe major depression	36714389	Depression	Depression	Conditions	
Severe major depression	42872722	Depression	Depression	Conditions	
Recurrent major depressive disorder with melancholic features	4205471	Depression	Depression	Conditions	
Major depression, melancholic type	4154391	Depression	Depression	Conditions	
Severe major depression without psychotic features	4327337	Depression	Depression	Conditions	
Moderate depression	4151170	Depression	Depression	Conditions	
Maternity blues	4133073	Depression	Depression	Conditions	
Drug-induced depressive state	4103126	Depression	Depression	Conditions	
Chronic recurrent major depressive disorder	4094358	Depression	Depression	Conditions	
Minimal major depression	36715000	Depression	Depression	Conditions	
O/E - depressed	4038252	Depression	Depression	Conditions	
Recurrent severe major depressive disorder co-occurrent with anxiety	35615152	Depression	Depression	Conditions	
Moderately severe depression	36717092	Depression	Depression	Conditions	
Recurrent moderate major depressive disorder co-occurrent with anxiety	35615153	Depression	Depression	Conditions	
Chronic major depressive disorder, single episode	4031328	Depression	Depression	Conditions	Excluded
Minimal depression	36713698	Depression	Depression	Conditions	
Depressive disorder in mother complicating pregnancy	37018656	Depression	Depression	Conditions	
Secondary dysthymia	4224639	Depression	Depression	Conditions	
Recurrent major depressive disorder in partial remission co-occurrent with anxiety	35615155	Depression	Depression	Conditions	Excluded
Postpartum depression	4239471	Postpartum depression	Depression	Conditions	
